# Evidence-Based Anti-Diabetic Properties of Plant from the Occitan Valleys of the Piedmont Alps

**DOI:** 10.3390/pharmaceutics14112371

**Published:** 2022-11-03

**Authors:** Valentina Boscaro, Matteo Rivoira, Barbara Sgorbini, Valentina Bordano, Francesca Dadone, Margherita Gallicchio, Aline Pons, Elisa Benetti, Arianna Carolina Rosa

**Affiliations:** 1 Dipartimento di Scienza e Tecnologia del Farmaco, University of Turin, Via Pietro Giuria 9, 10125 Turin, Italy; 2 Dipartimento di Studi Umanistici, University of Turin, Via Sant’Ottavio 20, 10124 Turin, Italy; 3 Atlante Linguistico Italiano (ALI), Via Sant’Ottavio 20, 10124 Turin, Italy

**Keywords:** diabetes, elderberry, yarrow, cornelian cherry, bilberry, wild strawberry, rosehip, raspberry, blackberry, stinging nettle

## Abstract

Data on urban and rural diabetes prevalence ratios show a significantly lower presence of diabetes in rural areas. Several bioactive compounds of plant origin are known to exert anti-diabetic properties. Interestingly, most of them naturally occur in different plants present in mountainous areas and are linked to traditions of herbal use. This review will aim to evaluate the last 10 years of evidence-based data on the potential anti-diabetic properties of 9 plants used in the Piedmont Alps (North-Western Italy) and identified through an ethnobotanical approach, based on the Occitan language minority of the Cuneo province (*Sambucus nigra* L., *Achillea millefolium* L., *Cornus mas* L., *Vaccinium myrtillus* L., *Fragaria vesca* L., *Rosa canina* L., *Rubus idaeus* L., *Rubus fruticosus/ulmifolius* L., *Urtica dioica* L.), where there is a long history of herbal remedies. The mechanism underlying the anti-hyperglycemic effects and the clinical evidence available are discussed. Overall, this review points to the possible use of these plants as preventive or add-on therapy in treating diabetes. However, studies of a single variety grown in the geographical area, with strict standardization and titration of all the active ingredients, are warranted before applying the WHO strategy 2014–2023.

## 1. Introduction

Diabetes mellitus is a chronic metabolic disorder characterized by persistent hyperglycemia [1]. The increased level of glucose in the blood damages body tissues over time, thus inducing the development of microvascular (including blindness, nephropathy, neuropathy and diabetic foot) and macrovascular (cardiovascular and stroke) complications that could be disabling and life-threatening. Unfortunately, diabetes prevalence is increasing globally, reaching epidemic proportions and represents a leading cause of death worldwide. Currently, about 538 million adult people are living with diabetes and by 2045 it is estimated that the number will increase to 783 million [2]. Nowadays, the economic burden associated with diabetes is very worrying, accounting for 12% of global healthcare expenditure [2,3] and these costs usually increase over time and with disease severity. Moreover, considering the expected increase in diabetes morbidity, the healthcare system expenditure on diabetes and its complications is bound to increase. Therefore, early investments into prevention and disease management are particularly worthwhile.

Accounting for over 90–95% of all cases, type 2 diabetes mellitus (T2DM) is the most common disease, thus representing the primary focus of diabetes research. The progressive impairment of insulin sensitivity results in insulin resistance, defined as an increased insulin requirement to maintain glucose homeostasis. This results from a reduced insulin ability to activate the insulin signaling pathway in the hormone-responsive tissue. Another diagnostic determinant of T2DM is pancreatic β-cell dysfunction. In the early stages of the disease, peripheral tissue responsiveness to circulating insulin is reduced and pancreatic β-cells increase insulin secretion to compensate for this insulin resistance. Over time, the ability of pancreatic β-cells to release sufficient insulin declines, resulting in impaired fasting glucose and impaired glucose tolerance associated with prediabetes. Further disease progression is characterized by continued β-cell deterioration and chronically elevated blood glucose concentrations [1,4]. Recognizing that the combinations of several unhealthy lifestyle factors play a crucial role in the pathogenesis of this disease, strategies focused on promoting an active lifestyle and a healthy diet are a cornerstone for preventing T2DM. One dietary factor of particular interest is the consumption of natural products. Several natural products, mostly plant-specialized (secondary) metabolites, have shown either insulin-mimetic or secretagogue properties, with a beneficial effect on glucose metabolism, thus suggesting that the consumption of these plants could lower the risk of T2DM [3,5,6]. These beneficial effects are mainly attributed to the content of different active ingredients, including alkaloids and terpenoids [7]. So far, in the literature, more than 400 plant species have been described as anti-hyperglycemic [8]. However, this definition raises several doubts and could appear quite superficial, because defining a substance as anti-hyperglycemic is a complex process requiring not only in vitro and in vivo evaluations, but above all clinical studies. Altogether, preclinical and clinical evaluations represent a two-step approach with the first suggesting and the second confirming the anti-diabetic effect. In vitro assays aim to assess the improvement in glucose uptake from insulin-dependent tissues, the modulation of insulin secretion and/or the possible inhibition of digestive enzymes such as α-glucosidase and α-amylase. In vivo measurements include fasting glycaemia, oral glucose tolerance test (OGTT), homeostatic model assessment for insulin resistance (HOMA-IR) and insulinemia. Positive results from these preliminary studies should be confirmed by a clinical evaluation based on the standard assays for the diagnosis of diabetes, such as glycosylated hemoglobin (HbA1c; ≥6.5%), fasting plasma glycaemia (≥126 mg/dL), 2 h OGTT (≥200 mg/dL) or random glycaemia (≥200 mg/dL) [9]. 

Focusing on natural remedies the experimental studies are not conclusive, mainly replicating previous results, without providing added value to the potential efficacy of the clinical application. Clinical trials are usually lacking and the few available include a relatively small number of subjects and/or have a relatively short-term endpoint. Therefore, the bench-to-bedside translation is very difficult and the contribution of natural remedies in real life is still an unresolved challenge. Despite the limits on the knowledge of natural remedies’ efficacy, reliance on them by the public is usually high as demonstrated by the wide use of this kind of product worldwide. This led to the investigation of the potential use of plants for different applications, including the most urgent therapeutic need area, as is ongoing with, for example, the proposed in vitro efficacy of *Sambucus nigra* L. [10,11], *Urtica dioica* L. [10,12], *Rasa canina* L. [13], *Rubus fructicosus* L. [14] or *Achillea millefolium* L. [15] against COVID-19 infection.

Consistently, promoting a culture of conscious use of these remedies is strictly urgent to avoid false efficacy expectations or, even worse, the onset of health issues. Diabetic patients have been reported to use herbal medicines [16] with an increase in their consumption of 380% in the US [17]. Therefore, a periodic review of the most recent literature on the anti-diabetic properties of herbal remedies allows for the addition of new pieces of evidence towards the conscious use of herbal remedies for the prevention/treatment of diabetes.

Interestingly, data comparing urban-to-rural diabetes prevalence ratios show a significantly lower presence of diabetes in rural areas [2] in Europe more than in other continents. According to epidemiological data in the Piedmont Region (North-Western Italy), the province of Cuneo (South-Western part of the Piedmont Region) is the area with the lowest prevalence of diabetes in the whole region, with a prevalence of diabetes in 2018 of 4.7% [18]. On the contrary, Turin (the capital city of Piedmont) registered in 2016–2018 a cumulative incidence of 11.5 × 1000 new diabetic patients with a prevalence of 5.9% [19]. Cuneo, the 19th Italian province by population, is covered more than half by mountains. The geographical-naturalistic heritage of Cuneo province, based on rural-artisan environments and small city settlements [20], explains the rural lifestyle of residents. Interestingly, a cross-sectional study in the mountainous region of Nepal demonstrated that diabetes mellitus prevalence is lower among residents at a higher altitude compared to the general population [21]. Therefore, it is possible to speculate that people living at a higher altitude in the Alps of Cuneo have a reduced risk of developing diabetes compared to people living in the same region’s plains. Moreover, residents could benefit from the specific Alpine biodiversity and ethnobotanical resources. The Piedmont Alps in Cuneo province show a peculiar heritage, as they belong to the Occitan-speaking valleys of Piedmont, a language minority of Alpine communities living 750 m above sea level (msl), with a solid herbal use tradition and agrarian economy tradition, beginning to be lost after the Second World War but recovering in the last decades [22], with the rediscovery of local traditions in favor of a return to agriculture flanked by tourism development. In the past years, the traditional knowledge of the plants of the Piedmont Alps was investigated through an ethnobotanical approach based on the concept of language and ethnic minority groups. Two papers, in particular, focused on the presence of several wild plants as famine foods and medicines in this region: the first [23] recorded 88 botanical taxa in the upper Val Varaita valley (Piedmont Alps, Cuneo Province), a quarter of them also known in other surrounding Occitan valleys, while the second [24] reported 92 plants belonging to 40 different families in three selected linguistic sites in the Alpine Occitan communities (Grana and Gesso valleys, both in the Piedmont Alps in Cuneo Province) [24]. These papers highlighted how different local folk uses of plants go far beyond their nutritional properties.

In the more general attempt to relocate wild alpine plants as functional foods (foods that have beneficial effects on one or more functions beyond their nutritional properties [25]) based on traditional knowledge, this review aims to analyze the spontaneous (not exclusive) presence of plants in the Piedmont Alps (Italy) used by the Occitan language minority in the Cuneo province, for which the literature showed in vitro and in vivo anti-diabetic properties and/or are subject to ongoing clinical evaluation for pre-diabetes and diabetes (Table 1). 

Included in this task, following an ethnobotanical analysis of the Piedmont Alps, a literature search of papers published between 2011–2021 was conducted using keywords including the botanical name of each plant, diabetes, T2DM, hyperglycemia and insulin resistance. Reviews, experimental and clinical studies were all considered. Finally, a search of clinical trials was performed on the ClinicalTrial.gov database. The review is structured in four sections: the first identifies and describes the selected plants in ethnobotanical terms; the second describes the main active ingredients with presumed anti-diabetic activity; the third, divided into sub-sections by single plant, provides a description of the experimental studies published in the 2011–2021-time frame; the fourth reports the results obtained from the most recent clinical studies. 

## 2. Plants from the Occitan Valleys of the Piedmont Alps and Their Traditional Use

Plants reported in Table 1 are all well known to the Piedmont Alpine Communities and their different local folk uses, far beyond their nutritional properties, are described below.

Still, no reference to anti-diabetic properties has been found in the traditional knowledge of the Occitan valleys of the Piedmont Alps. 

Among plants belonging to the Rosaceae Family, different well-known species have been identified (Table 1); most of them are used for their delicious fruits as food but also present exciting folk uses in the Piedmont region. The Rubus genus is the largest genus of the Rosaceae family, distributed all over the world except Antarctica [26,27]. It comprises more than 750 species and 12 subgenera [26]. The species of this genus have been cultivated for centuries for their fruits, which are consumed fresh or as processed products [28,29]. 

### 2.1. Rubus idaeus L. (Raspberry)

*Rubus idaeus* L., or raspberry, belongs to the ampula type: according to some, the name is based on a pre-Indo-European base amp-; according to others, it is instead a continuer of a Latin ampulla form due to the shape of the fruit once detached from the peduncle which has a deep cavity inside [30,31,32]. Indeed, the pendulous fruits are aggregates of different berries, first green and then red when ripe (drupetum). Raspberry is a perennial shrub characterized by a creeping rhizome from which branch green or reddish-brown stems up to 2 m high. Stems are woody, pruinose, glabrous, sometimes with simple hairs and small patented or inclined spines, sometimes violet-colored. The flowers are hermaphroditic and grouped in inflorescences of up to 10 each; the calyx is composed of five persistent, triangular, reflexed, whitish-grey sepals. Petals are equal in number to the sepals, deciduous, white and shorter than the sepals. The leaves are deciduous, characterized by an upper light green page and a lower whitish, a petiole and a spiny-tomentose rachis. They are pinnate with three to seven ovate-lanceolate leaflets sprinkled with very short simple or star-shaped hairs, very variable in size and shape, with serrated margins [33]. EMA recognizes the traditional use of raspberry leaf, whose minimum content of tannins should be 3% according to the European Pharmacopoeia [34,35], for minor spasms associated with menstrual periods, for the symptomatic treatment of mild inflammation in the mouth or throat and the symptomatic treatment of mild diarrhea. The fruits of red raspberry can be consumed in raw or processed forms (frozen, dried, juiced, powdered) or extracted [36]. The information on the use of its fruits in the Occitan valleys is sparse but covers a wide range, from jam to syrup or liqueur [37,38]. In eastern Europe, raspberry fruits have also been used to treat the common cold, fever and flu-like infections [27]. These uses can be ascribed to the presence of several phenolic compounds, the predominant being anthocyanins (around 92.1 ± 19.7 mg anthocyanins/100 g of fresh fruit, although variability between fruit variety, season, developmental stage and methods to quantify the compounds have been reported [39]) and ellagitannins such as sanguiin H-6 (from 139.2 ± 14.4 mg/100 g dry weight to 633.1 ± 65.6 mg/100 g dry weight according to the different variety) and ellagic acid (from 26.1 ± 2.6 mg/100 g dry weight to 106.8 ± 10.9 mg/100 g dry weight according to the different varieties) [40]. Sanguiin H-6 activities have been tested in different in vitro experimental settings and extensively reviewed [41]. According to the values reported by the authors, the anti-inflammatory and anti-oxidant effects have been achieved in a range of concentrations between 2.5 and 250 µM, depending on the specific assay. Moreover, sanguiin H-6 showed a minimum inhibitory concentration (MIC) between 0.06 mg/mL (against *Clostridium sporogenes*) and 0.5 mg/mL (for *Streptococcus A*, *Pneumoniae*, *Bacillus subtilis*, *Moraxella catarrhalis*); on the contrary, the MIC calculated for *Candida albicans* is 5 mg/mL [41]. 

### 2.2. Rubus fructicosus L./Rubus ulmifolius (Blackberry)

*Rubus fruticosus* L. and *Rubus ulmifolius* Schott (blackberry) are other widely used mountain plants as folk remedies. The lexical type prevalent in the western Alps is *moura* with explicit reference to the dark color of the fruits [30], well known for being a functional food [29]. *Rubus ulmifolius* Schott is a perennial sarmentose shrub growing in almost all of Europe, North Africa and Southern Asia; it has also been introduced in America and Oceania. Shrubs present aerial stems characterized by a pentagonal section, up to 6 m long, provided with curved thorns. Leaves are dark green, quite persistent, ternate to palmate with stipules filiform to linear; leaflets are three to five, with a base rounded to cuneate, margins finely to moderately serrate, apex acute or acuminate. Flowers are white or pink with five petals and five sepals. They are grouped in racemes with 10 to 60 bisexual flowers forming oblong or pyramidal inflorescences. The edible fruit is composed of numerous small drupes (generally 10 to 40), green at first, then red and finally blackish when ripe, each arising from separate carpels but forming part of the same gynoecium [42]. Blackberries have a high content of carbohydrates, vitamins (ascorbic acid in particular), minerals (potassium included) and dietary fiber suggesting their use in diet-based therapies for improving human health. They are also rich in specialized bioactive metabolites, mainly phenolic compounds and ellagitannins, which may contribute to the anti-oxidant and anti-inflammatory properties of these berries [26,28,43,44]. In Italian folk medicine, blackberry treats ulcers, intestinal inflammations, diarrhea, abscesses, furuncles, red eyes, vaginal disorders and hemorrhoids [45]; it is also used as an anti-pyretic and carminative agent [26]. A crude methanolic extract and flavonoid fractions obtained from dried materials of aerial parts of blackberry showed significant anti-oxidant and anti-pyretic activity in rats with pyrexia [26]. The effect on the gastrointestinal tract can be due to the biological activity of polyphenols, with blackberry improving gastrointestinal digestion and protecting against ethyl carbamate and acrylamide-induced oxidative stress and cytotoxicity [46]. However, the primary folk use in the Occitan valleys recalls mostly the food consumption of blackberry fruits, with the preparation of jams. As a folk remedy, some evidence focused on its use for sore throats: a syrup prepared from the blackberries cooked, passed through a sieve to remove the small grains and cooked again with sugar and a gargling solution after infusion of spring jets, has been described [37,38]. Blackberry’s use for sore throats or whooping cough stems from its anti-microbial, anti-oxidant and anti-inflammatory effects. Suit and leaves have a lower anti-microbial activity compared to root or stem [47]. The leaves are also rich in tannins and used to counteract diarrhea or, when used externally, for wound healing [48].

### 2.3. Fragraria vesca L. (Wild Strawberry)

*Fragaria vesca* L. (wild strawberry) always belongs to the Rosaceae family. The rugged woodland strawberry is native to Europe and Asia. It is the most widely distributed species of *Fragaria* occurring throughout Europe, Northern Asia, North America and Northern Africa. It is an herbaceous perennial plant characterized by an oblique and branched rhizome and short, slender, weakly woody stems. It reaches a height ranging from 5 to 20 cm. The trifoliate leaves are as long as the scape, with a long, pubescent petiole; the upper page is dark green, while the lower is whitish, slightly hairy, especially on the nerves; the margin is roughly toothed. The scape is characterized by one to three erect flowers, becoming pendulous at the fruiting stage; flowers have a corolla composed of five white petals with overlapping margins, triangular sepals and 20 stamens. Fruits are small, black achenes scattered on the swollen red receptacle, easily detached from the calyx [33,49]. Wild strawberry is reported to have two basic lexical types in the western Alpine valleys: *frola-frùi* and *maiòla-maiùso*, sometimes with an alternative suffix [30]. EMA recognizes in wild strawberry leaf its traditional use to increase urine production in patients with minor urinary tract problems and to relieve symptoms of mild diarrhea [35]. Moreover, its leaves were reported to have healing properties when applied to the top of cuts or wounds [50]. These effects can be due to all the active ingredients present in wild strawberry leaves, including phenolic compounds (mainly ellagitannins, flavonoids, proanthocyanins, in particular catechins, phenolic acids), terpenes characterizing volatile oils (e.g., borneol), methyl salicylate and trace amounts of alkaloids [51,52]. The plant is rich in vitamin C and folate [5]. Therefore, its extract-based hydrogel at 2% has been recently studied for topical cosmetic applications [53]. 

### 2.4. Rosa canina L. (Rosehip)

The last plant in the Rosaceae family approached in this review is *Rosa canina* L. (rosehip or dog rose, Table 1). In the western Occitan, there are three main lexical types of rosehips. The first, also in Piedmont, is ‘*grattaculo*’, no longer diversifying between the plant’s name and the fruit. In this case, the etymological process proposed goes back to the name *crataeugus*, reinterpreted as *grattaculo.* The second is *agoulenchìer*, widespread in the high valleys and deriving from *aquilentum* crossed with *aculeus*, concerning the thorniness of the shrub. The third type collects those names that refer to the appearance of the plant (bush, presence of thorns). In some cases, the fruit is distinguished from the shrub *bossou*, a name elsewhere referring to the wild blackthorn [30]. It is a shrub-like species growing spontaneously throughout Europe. A woody, bushy and thorny deciduous shrub, its deep roots and pendulous branches covered with strong, bent, or hooked thorns, mainly with a swollen base, characterize it. The leaves are imparipinnate, with 2 lanceolate stipules at the base; leaflets are soft green, oval, or ovate-elliptical, usually glabrous, or slightly pubescent on the rachis, without glandular hairs. The flowers are solitary or in groups of two to three, delicately scented; corolla has five white or pinkish petals and five green concrescent sepals that fall off when the fruit ripens. The false (or pseudo) fruits, called cinorrods, ripen in autumn and are pyriform, fleshy and glabrous, usually borne by 10–20 mm long peduncles, bright red when ripe. The cinorrods contain many hard achenes covered with short, stiff hairs [33,49]. The cinorrods and petals are mainly used to flavor grappa, produce a sour jam or a sauce to accompany meats, or as a sort of bread by grinding the internal seeds. Moreover, harvested and dried cinorrods are used in herbal tea for their vitamin and diuretic properties. The volume *Piante e Fiori delle nostre Montagne* [54] reports that “the leaves have contrasting properties: both anti-diarrheal and mild laxative. The petals are corrective, refreshing, laxative, anti-diarrheal and flavoring and used externally in eye drops, mouthwashes, or washing; the rosehips are used as corrective, refreshing vitamins due to the high concentration of vitamin C, anti-inflammatory for the genitourinary system, diuretics, or astringents. The external use of pseudo-fruits includes toning, lightening and smoothing of the skin, or for their anti-hemorrhoidal properties. They are also prepared in preserves and jams. Some indicate the seeds as relief for mild symptoms of mental stress and as wormers against roundworms. Galls […] have diuretic and anti-perspiration activity; they are astringent and decongestant for external use. Galls were considered possible substitutes for ergot as regulators of uterine contractions”. Consistently, different medical uses can be identified in folk medicine: decoctions for the treatment of bronchitis and cough, for liver stones and to regulate blood pressure [31]; the pseudo-fruit was used as food in times of famine [55]; the herbal tea was used to fight colds, or to treat inflammation of the gums, especially in children, as “pink honey” (prepared cooking rosehip petals with apples). Finally, the use of flower petals to make healing compresses for the eyes should be noted. All these effects reflect the identification of numerous bioactive compounds, including vitamin C, flavonoids, tocopherols, carotenoids, organic acids, sugars and essential fatty acids [56,57]. Rosehip, made up of the receptacle and the remains of the dried sepals of rosehip, should have a minimum vitamin C content of 0.3% (dried drug) according to the European Pharmacopoeia [34]. The anti-inflammatory properties have been generally attributed to linoleic acid, α-linolenic acid and, more importantly, the galactolipid (2S)-1,2-di-O-[(9Z,12Z,15Z)-octadeca-9-12-15-trienoyl]-3-O-β-d-galactopyranosyl glycerol (GOPO). This latter active ingredient, isolated from seeds and fruits, reduced the chemotaxis of peripheral blood polymorphonuclear leukocytes, neutrophils, monocytes and C-reactive protein levels [58]. Consistently, preliminary clinical data showed its efficacy in reducing the complaints of patients with osteoarthritis, thus improving their quality of life [59]. 

### 2.5. Vaccinum myrtillus L. (Bilberry)

Well known to Alpine communities, *Vaccinium myrtillus* L. (bilberry) is a species belonging to the *Vaccinium* genus and the Ericaceae family. It belongs to a large genus: blueberry (*Vaccinium corymbosum*) and cranberry (*Vaccinium macrocarpon*). Several names are lexical types that refer to bilberry, also known as the European blueberry, whortleberry, huckleberry and blueberry. In the Western Alps, the most common types generally refer to the color of the berries, e.g., Latin *ater* ‘dark’ *aidre*, *arëzze*, *aize* (types of the Occitan valleys; for *areze*/*erze,* other proposals have also been made: *acinus* + *ater*, *acris* or *prel*. * *alisa*) or their shape, e.g., *prelatin* * *brom*- ‘rounded object’ *ambruna* (type of the Franco-Provençal valleys) [31]. Bilberry is a low-growing (between 10 and 40 cm) perennial shrub in North Europe, North America and Asia. Bilberry has small alternate leaves, green on both sides, characterized by a thin oval or elliptical lamina and short petioles. The flowers are hermaphrodite, actinomorphic with 4–7 mm reflexed pedicels, usually reddened. The calyx is gamosepalous and divided into five very short obtuse lobes. The corolla is greenish-white or pinkish with 5 small, revolute lobes. Fruits are small berries (5–9 mm in diameter), blue-violet or blackish, covered by bloom and slightly flattened at the apex. The inner part is paler and contains numerous brown seeds [33]. The flavor is acidulous and pleasant. Therefore, fruits have been used in various culinary preparations, fresh or jam, for a long time. Nevertheless, thanks to the high content in anthocyanins, mainly in the glycosylated forms (cyanidin-, delphinidin- and malvidin-3-O-glycosides), vitamins, catechins, phenolic acid derivatives and minerals [60], several beneficial effects have been reported for bilberry. Fresh fruits have a laxative effect, while dried or as a juice they have an astringent effect. The leaves have anti-inflammatory properties and are used for oral, skin, or ocular inflammation, thanks to anthocyanins. Its anti-thrombotic, anti-hypertensive and cardioprotective properties are well known [60], for which not only fruits but also leaves were used [31]. Notably, relieving discomfort and heaviness of legs related to minor venous circulatory disturbances or symptoms of bleeding into the skin from tiny blood vessels are traditionally recognized by EMA for fresh fruit. As for dried fruit, EMA recognizes traditional use for treating mild diarrhea and minor inflammations of the oral mucosa [35]. Both fresh and dried fruits are described in the European Pharmacopoeia, with a minimum content of 0.30% of anthocyanins, expressed as cyanidin 3-O-glucoside chloride and 0.8% of tannins, defined as pyrogallol, respectively [34]. Among other plant uses, boiled leaves were administered to newly relieved cows, facilitating the expulsion of the placenta [55]. 

### 2.6. Sambucus nigra L. (Black Elderberry)

*Sambucus nigra* L. (black elderberry or elder, elderberry, European elder, European elderberry and European black elderberry) is a species belonging to the Adoxaceae family, known in the Western Italian valleys as sambuca according to the primary Italian lexical type, which continues the Latin *Sambucus*. Also from Latin, *Sambucus nigra* L. is also referred to as *seuic*. It is a small tree or shrub not exceeding 5 m in height, growing native in Europe, northern Africa and southwestern Asia. It is characterized by a gray bark and numerous protruding lenticels. The leaves, bright green in color, are opposite, pinnate with 5–7 leaflets, oval or oblong, acuminate and with irregularly serrated margins. The odorous tiny white flowers are grouped in large corymb inflorescences; they are actinomorphic and are characterized by a rounded corolla composed of five ivory white, sometimes reddish, oval petals. The fruits are small globular drupes, first green, then purple-blackish, shiny and juicy when ripe, containing two to five oval, brown seeds. Fruits are grouped in pendulous infructescence on reddish peduncles [49]. Historically the whole plant was used, from the wood to the flowers and the fruits, thanks to the high content of carbohydrates, proteins, fats, fatty acids, organic acids, minerals, vitamins, essential oils and anti-oxidants such as polyphenols [61]. The anti-oxidant activity explains the use of elderberry fruits and flowers in different therapeutic contexts, including toothache, colds and udder diseases. It is used as a refreshing and purifying agent and as an anti-hypertensive remedy, also in animals. The leaves were used against rheumatism [31]. “In medicine, the flowers of the plant are prepared as compresses, attenuate swellings of various origins and, in herbal tea, calm toothache” [55]. Moreover, infusion of dried flowers was used against colds and bronchitis; the fruits, in sweetened juice or jams, as a mild laxative and against neuralgia [37,38]. EMA recognizes the traditional use of elderberry flowers to relieve early symptoms of common cold [35]. The minimum content of flavonoids should be 0.80%, expressed as isoquercitroside, according to the European Pharmacopoeia [34]. 

### 2.7. Achillea millefolium L. (Yarrow)

Much more widely known and used in the mountain areas than in the municipalities of the plains [37,38] is *Achillea millefolium* L. (yarrow), a species belonging to the Compositae family. It is an aromatic perennial herbaceous plant, rhizomatous and slightly suffruticose, covered with hair or down, 4–90 cm high. Leaves are alternate, oblong-lanceolate, green or greyish green, slightly pubescent on the upper surface and more pubescent on the lower surface, two–three pinnatisects divided into linear hairy segments. The flowering tops containing essential oil, also described in the European Pharmacopoeia, are the most active part of the plant [62,63]. Flower heads are white or pinkish and are arranged in corymbs at the end of the stem. Each flower head consists of a slightly convex receptacle, 4–5 ligulate ray florets and 3–20 tubular disk florets. Fruits are 2-mm shiny achenes with no pappus [33]. The lexical type prevalent in the Western Alps is cut grass, *taiouira* or *mal tagliata* “badly cut,” due to its hemostatic properties and perhaps also in connection with mowing practices. Disinfectant and anti-inflammatory uses are also known. With local specific preparations and indications, it was used as a sedative in poultices for sprains and dislocations; decoctions were used against hemorrhoids and abdominal pain; in packs and infusions for hemorrhoids, flowering tops were collected to prepare digestive, calming and pain-relieving effects, in oil with camphor for muscle massage or whole plant to make compresses for toothache [31]. In other parts of Italy, yarrow is used for a variety of conditions, in particular gastro-intestinally [64,65]. Its use is also reported in Peru for gastritis, diabetes, cholesterol and mainly for skin infections [66]. Consistently with these local uses, EMA considers herbs and flowers as traditional herbal medicinal products for temporary loss of appetite and for the symptomatic treatment of mild, spasmodic gastro-intestinal complaints, including bloating flatulence or minor spasms associated with menstrual periods [35]. Moreover, they can be used as a traditional herbal medicine to treat minor superficial wounds (a leaf pack was also used as wound healing [31]). It can be found in herbal pharmacies as tinctures and capsules containing dry flowers or aerial parts [67]. Apart from the described folk uses, pleiotropic effects, including anti-diabetic, anti-tumor, anti-fungal, anti-septic and liver protective effects, have been suggested. These effects have been mostly proven in vitro, on isolated organs, or in vivo, but to the best of our knowledge not in the clinic; thus, their transferability to the real world is far from being demonstrated. The reason for this plethora of potential effects stems from the several active ingredients of yarrow including phenols (such as choline, caffeoylquinic acids (DCCAs) or salicylic acid), flavonoids (including apigenin, artemetin, luteolin-7-O-β-D-glucuronide, dihydro-dehydro-diconiferyl alcohol 9-O-β-D-glucopyranoside) and many monoterpenes (such as borneol, camphor, eucalyptol, α- and β-pinene, α-terpineol and sesquiterpenes (chamazulene, azulene, included), with monoterpenes constituting 90% of the essential oils compared to sesquiterpenes [63,67,68,69,70,71,72,73,74]. Actually, essential oil is the only feature reported by the European Pharmacopeia, according to which its minimum content in whole or cut, flowering tops should be 2 mL/kg dried drug [34]. The essential oil is the formulation mostly related to the folk uses described above. Other uses have been suggested for different herbal preparations, such as decoction, hydroalcoholic, methanolic and aqueous extract [75], in which specific active ingredients can be recognized. For instance, a choleretic activity has been ascribed to teas or tinctures, for their content in luteolin-7-O-β-D-glucuronide and DCCAs, whose activity was demonstrated in isolated organ experiments [67,76]. The hepatoprotective effect was tested in vivo with ethanol [77], aqueous [78], or aqueous–methanol [79] extract from the aerial parts of the plant. The active ingredient detrimental to this effect has not yet been clearly established, although some evidence points out at the 5-hydroxy 3, 4′, 6, 7-tetramethoxy flavone [78]. The chloroform extract, in which flavonoids (apigenin, luteolin, centaureidin, casticin and artemetin) and sesquiterpenoids (paulitin, isopaulitin, psilostachyin C, desacetylmatricarin and sintenin) are present, showed in vitro antiproliferative activities [67].

### 2.8. Urtica dioica L. (Stinging Nettle)

*Urtica dioica* L. (stinging nettle) is a well-known plant with the same lexotype (lat. *Urtica*, meaning “to burn,” attributed to its stinging hair) with different phonetic results [30]. The specific name *dioica* is related to the fact that it is a dioecious plant, having unisexual flowers (male or female) on different plants. Nettle is an herbaceous perennial plant growing worldwide. It is characterized by long stoloniferous rhizomes branched just below the surface, from which numerous robust, erect and striped quadrangular stems arise. Stems are covered with unicellular stinging hairs. The leaves are opposite, with a petiole shorter than the leaf blade (not reaching its midpoint); they are much longer than wide, ovate-lanceolate, with a heart-shaped base and acute apex; the margin is toothed. In addition, leaves are covered by long stinging hair together with short, simple hairs. The small flowers are grouped in the glomerulus, greenish-yellow or reddish, inserted at the leaf’s axil. The fruit is an ovoid-elliptic, olive-brown diclesium, with a tuft of hairs at the apex, enclosed in the enlarged petals [33]. Stinging hairs contain several chemicals, including serotonin, histamine, acetylcholine, moroidin, leukotrienes and formic acid, that provoke pain, wheals, or a stinging sensation. However, the irritant power is lost during cooking, thus making this plant usable for culinary purposes, consistently with its high content in nutrients (up to 3.7% proteins, 0.6% fat, 2.1% ash, 6.4% dietary fiber and 7.1% carbohydrates in harvested upgrowths [80], high concentrations of vitamins and metals in fresh leaves [81] and about 30% proteins, 4% fats, 40% non-nitrogen compounds, 10% fiber and 15% ash in leaf powders [82]), it is appreciated as food in general: stinging nettle tips are the first to be harvested in spring. They are used in omelets or herb soups and risotto. Besides several nutrients, stinging nettle is rich in biologically-active compounds such as terpenoids [83], carotenoids, fatty acids, polyphenols [81,84,85,86], tannins, carbohydrates, sterols, polysaccharides, isolectins and minerals, the most important of which is iron [82]. The distribution of phytochemicals is uneven: leaves are rich in flavonoids (mainly quercetin, kaempferol, rutin and their 3-rutinosides and 3-glycosides, catechin, epicatechin and epigallocatechin-gallate); root contains lectins, polysaccharides, phytosterols, lignans, coumarins (scopoletin) and high amounts of fatty acids; nettles have abundant quantitative of flavonoids, in particular, anthocyanins; flowers contain high amounts of β-sitosterol and 7-flavonoid glycosides [87,88,89]. The European Pharmacopoeia describes only leaves and radix [34]. EMA recognizes the traditional use of the leaves and herbs to relieve minor articular pain and increase the amount of urine and as an adjuvant in minor urinary complaints. Herbs could also be used for seborrheic skin conditions [35]. The radix was traditionally used to relieve lower urinary tract symptoms related to benign prostatic hyperplasia. These uses are like stinging nettles by local populations in Piedmont, where also veterinary uses are described. In humans, it was applied as anti-anemic due to its content in iron, a digestive and purifying agent, against bruises and hair loss. Stinging nettle broth was used for ulcers and cosmetic applications, especially against acne or greasy hair. Finally, folk uses included the treatment of sciatica and rheumatism, as well as diuretic or metabolic effects. In cows, stinging nettles were used to treat diarrhea and in hens to promote the production of eggs (it is considered “warming”). The other best-known use of stinging nettle leaf macerate is against plant lice, possibly leaving it to macerate with tobacco [50].

### 2.9. Cornus mas L. (Cornelian Cherry)

Less used because not always known to local communities is *Cornus mas* L., cornelian cherry or dogwoods. It is a sporadic species in the Western valleys belonging to the Cornaceae family. The primary lexical type is etymological to the Italian: *cournal*. Cornelian cherry is a deciduous shrub that can grow up to 6–8 m in height. The trunk is erect, often twisted, highly branched at the top, gray with reddish cracks, peeling bark and short erect-patent twigs. The leaves, with a short hairy petiole, are oval, opposite and acuminate. The small yellow flowers bloom before the leaves and give off a faint honey smell; they are grouped in axillary umbels, surrounded by four acuminate greenish bracts. The calyx is composed of four acute greenish sepals, while the corolla by four acute, glabrous, golden yellow petals. The fruits are ovoid edible dark red drupes [33]. Records of its use come from mountain or foothills villages. Healing decoctions and sour jam were prepared from the fruits, characterized by local anti-inflammatory properties, due to the content of anti-oxidants such as phenolic metabolites, concerning anthocyanins [31,90]. 

## 3. Active Ingredients of Plants from the Occitan Valleys of the Piedmont Alps Recognized to Have Anti-Diabetic Properties: An Overview 

The Alpine plants identified in our study are characterized by common active ingredients justifying the use of these plants as functional foods or nutraceuticals. However, each plant’s percentage of active ingredients varies according to its species and genus. Therefore, despite the coexistence of different active ingredients pointing out a possible synergism, the anti-diabetic effects can be mostly attributed to some active ingredient classes such as polyphenols, anthocyanins in particular and oligosaccharides (Table 2). Their relevance relates to their direct effect on signaling pathways involved in glucose homeostasis. 

### 3.1. Poliphenols

The most commonly represented active ingredients in the plants considered are polyphenols. Polyphenols represent a large chemical class of natural compounds, with one or more hydroxyl groups attached to the aromatic ring [138], that share the ability to act as anti-oxidants, thus protecting against the damage caused by oxygen-free radicals to DNA and several cellular components [139]. The polyphenols could be classified into different groups by the phenol ring number and the structural elements connected to the ring [140]. Usually, a first distinction is between flavonoids (divided into anthocyanins, flavonols and flavanols based on the oxidation state of the heterocyclic pyran ring) and non-flavonoids (such as phenolic acid, including benzoic acids and hydroxycinnamic acids, stilbenes and its derivative, including gallotannins, ellagitannins and lignins) [141].

It is challenging to dissect each polyphenol’s relative contribution to glucose metabolism, but polyphenols are usually evaluated for their anti-diabetic potential. Therefore, several mechanisms could explain their role in glucose metabolism: they reduce glucose absorption, increase glucose metabolism and pancreatic insulin secretion and enhance glucose use by muscle and adipocytes. Polyphenols such as flavonoids and tannins inhibit critical enzymes involved in the digestion of carbohydrates to glucose, such as α-glucosidase and α-amylase [142,143]. Polyphenols can regulate postprandial glycemia by a facilitated insulin response and the release of the glucose-dependent insulinotropic polypeptide (GIP) and glucagon-like polypeptide-1 (GLP-1) [144,145]. Glycolysis, glycogenesis and gluconeogenesis, generally impaired in diabetes, could be controlled by polyphenols; for example, epigallocatechin gallate, naringenin and hesperidin increased the expression of glucokinase (GK) at hepatic level [146,147] and naringenin decreases the expression of glucose-6-phosphatase. Moreover, polyphenols such as resveratrol, quercetin and epigallocatechin gallate can enhance the translocation of glucose transporter (GLUT)4 to the plasma membrane, thus increasing glucose uptake in muscle and adipocytes by the activation of the AMP-activated protein kinase (AMPK) pathway [148], which is a sensor of the cell’s energy that plays a vital role in obesity. Polyphenols could suppress β-cell damage induced by prolonged exposure to high glucose concentrations, for example, by decreasing oxidative stress. Anthocyanins could protect β-cells from oxidative stress affecting phosphoinositide 3-kinases (PI3K)/protein kinase B (AKT) and extracellular signal-regulated kinase (ERK)1/2 pathways [148]. Other polyphenols could upregulate essential genes involved in β-cell functions, such as *Glut2*, *insulin* (*ins*)*1*, glucokinase gene (*Gck*) *and sirt1* [149]. Catechins increase insulin release upregulating the expression of the nuclear factor erythroid 2–related factor (*Nrf*)*2* and *1* gene [150]. Moreover, polyphenols can block inflammatory pathways by inhibiting several protein kinases overexpressed in diabetes [139].

Among polyphenols, anthocyanins are the largest group of water-soluble vacuolar polyphenolic compounds [151,152,153] in the plant kingdom with a positive charge in acidic solution; they are responsible for the characteristic red to blue color of plants [154] and are primarily present in fruits and flowers. These molecules consist of an anthocyanidin “core” with a sugar moiety (glucose, galactose, xylose, arabinose, or rhamnose) attached at various positions [151,155,156]. Hydroxyl and methoxyl groups can be attached to the core anthocyanin in different numbers and positions. These compounds are cyanidin, delphinidin, malvidin, pelargonidin, peonidin and petunidin. The primary sources of anthocyanins, apart from vegetables such as red cabbage and radish, are dark fruits such as blackberry, blueberry, cranberries, black and red currants, red grapes and raspberries. Accordingly, plants rich in anthocyanins, bilberry, blackberry and raspberry. are collected in natural environments in the Alps. Their content in anthocyanins varies across species and depends on environmental factors, such as solar radiation, temperature, soil content of nitrogen and phosphorus and the cultivation technique. Bilberry plants located at high latitudes or altitude have been demonstrated to produce berries with higher contents of anthocyanins [157]. Several studies demonstrated that anthocyanin amount could be particularly affected by processing. Frozen strawberries, blueberries and raspberries showed an average 42% decrease in anthocyanin content in their raw forms; the juice of berries retains only 22% of anthocyanins contents compared to their raw whole fruit form [36]. Storage temperature and heat treatment may degrade anthocyanins more than other flavonoids [158], while freeze-drying seems the most effective way to preserve anthocyanins in strawberries [159]. Anthocyanins have anti-oxidant effects causing the increased synthesis of endogenous anti-oxidant enzymes such as glutathione peroxidase (GPx), catalase (CAT) and superoxide dismutase (SOD) and exert different properties, such as DNA stabilization and adipocyte gene expression modification; they also have anti-apoptotic, anti-inflammatory and anti-bacterial effects. However, like other anti-oxidants, anthocyanins can produce a prooxidant effect in very high doses, not practically achievable via the diet, so the dietary intake of anthocyanins poses no concern. Numerous epidemiological studies demonstrated that a higher intake of total flavonoids, especially anthocyanins, could be associated with a lower risk of hypertension, myocardial infarction, stroke and T2DM [160,161,162,163]. The amelioration of insulin sensitivity could be explained through different mechanisms, such as the upregulation of GLUT4 gene expression in white adipose tissue and skeletal muscles [164], the activation of AMPK, which stimulates glucose uptake and insulin secretion by pancreatic β-cells. AMPK activation could also downregulate gluconeogenesis in the liver [165,166]. Anthocyanins could also regulate postprandial glycemia. Berries can reduce glucose absorption from a meal and inhibit α-glucosidase and pancreatic α-amylase activity, thus decreasing glycemia after starch-rich meals [167,168]. The mechanism of α-glucosidase inhibition action by anthocyanins is not fully understood. Still, one can assume that acarbose is a competitive inhibitor whose activity is due to the structural similarity between normal substrate maltose and the glucosyl groups β-linked to the anthocyanin. Therefore, anthocyanins bind to the enzyme’s active site without undergoing hydrolysis [169]. Moreover, anthocyanins significantly reduced the blood glucose level in diabetic rats, increasing the expression and translocation of GLUT4 and enhancing the activation of the insulin receptor phosphorylation, thereby increasing the uptake and utilization of glucose by cells [160]. Anthocyanins also improve insulin secretion, which could be significant for people with T2DM whose pancreatic activity is damaged. 

### 3.2. Oligosaccharides

Another class of active ingredients represented in the selected plants is oligosaccharides. Oligosaccharides, physiologically active fragments of plant cell wall polysaccharides, are low molecular weight polymers usually composed of 2 to 10 monosaccharides linked by glycosidic bonds non-susceptible to human digestive enzymes [170]. Of both natural and chemical, physical, or biochemical origin, the plethora of oligosaccharides includes functional compounds such as fosfo-fructo-oligosaccharides (FOSs), galacto-oligosaccharides (GOSs), mannan oligosaccharides (MOSs), xylo-oligosaccharides (XOS), neo-agaro-oligosaccharides, iso-malto-oligosaccharides and chito-oligosaccharides (COSs), used as prebiotic [171]. All oligosaccharides have the potential for prebiotic action. So far, the first hypothesis for their role in improving glycemic control and reducing the risk of developing diabetes could be based on their ability to improve the intestinal microbiota, which in turn stimulates insulin signal [172], for instance, increasing gut hormones including GLP-1 and peptide YY (PYY) [173].

Moreover, prebiotics have been demonstrated to inhibit intestinal α-glucosidase, α-amylase and glucose transporters, improve insulin resistance, glycogen synthesis and gluconeogenesis, affect the AMPK pathway in the liver and reduce the inflammatory status associated with diabetes and reduce circulating lipopolysaccharide (LPS), interleukin-6 (IL-6), monocyte chemoattractant protein-1 (MCP-1) and tumor necrosis factor-α (TNF-α). All these events have been long studied and extensively reported for the functional oligosaccharides, which means depolymerized or synthetic polymers used as prebiotics [173]. Compared to functional oligosaccharides, natural oligosaccharides occurring in plants were less studied, although at the mechanistic level they are supposed to exert several effects, including immune-stimulation and anti-oxidant effects, also by specific linkage of a glycan sequence and cell receptors [174].

## 4. Mechanistic Interpretation of the Anti-Diabetic Effects of Plants from the Occitan Valleys of the Piedmont Alps: Evidence from Experimental Studies

As the identified Alpine plants have many similarities in their active ingredient composition, they can share many anti-diabetic mechanisms. However, each mechanism could be more or less evident based on the percentage of each active ingredient. Table 2 summarizes the anti-diabetic mechanism(s) proposed for each plant according to the experimental studies published between 2011 and 2021. 

**Figure 1:**
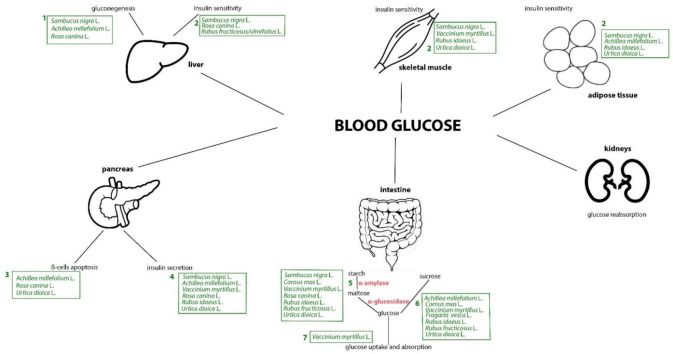
Plants used in the Occitan valleys of the Piedmont Alps: target tissues of their anti-diabetic effects. The selected plants have been suggested to reduce blood glucose levels by acting on various organs and tissues involved in glucose homeostasis. According to the experimental evidence published between 2011 and 2021 they can be grouped into plants promoting: 1 = gluconeogenesis downregulation; 2 = insulin sensitivity increase in liver, skeletal muscle and adipose tissue; 3 = islet atrophy inhibition and/or pancreatic β-cells regeneration promotion (associated with a reduction of inflammatory disease and increased proliferation); 4 = insulin secretion from pancreatic β-cells; 5 = α-amylase inhibition; 6 = α-glucosidase inhibition; 7 = reduction of glucose uptake from the intestinal epithelium.

Three events are triggered: (1) insulin secretion, (2) insulin sensitivity and (3) intestinal glucose absorption (Figure 1). 

The evidence of these mechanisms for each plant relies on a different number of studies, with stinging nettle one of the most extensively studied. 

### 4.1. Stinging Nettle

Stinging nettle’s hypoglycemic properties have been known for centuries, since the time of Avicenna [175]. In recent decades, several studies have investigated its anti-diabetic effects and the related mechanism of action. All the three events mentioned above have been demonstrated. These effects are ascribed to hydroxycinnamic acid derivatives and flavonoids, quercetin, in particular, being the central bioactive molecule of the aqueous extract [176]. Interestingly, the aqueous extract is the most effective in counteracting hyperglycemia compared to the hexane, chloroform, ethyl acetate, or methanol extracts [92].

**Figure 2:**
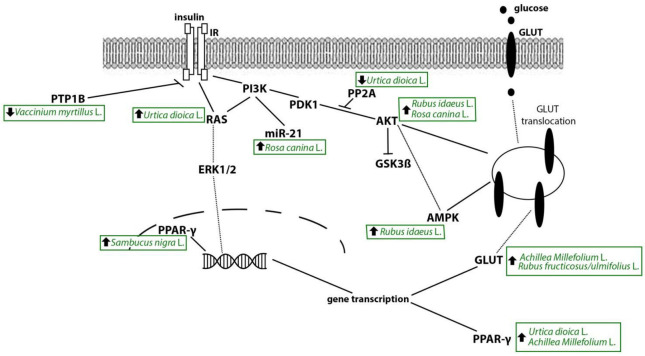
Plants used in the Occitan valleys of the Piedmont Alps: activation of the insulin signaling. According to the experimental evidence published between 2011 and 2021, the selected plants stimulate insulin signaling at different molecular levels. Their principal molecular targets are schematically represented. IR: insulin receptor; GLUT: glucose transporter; PTP1B: protein tyrosine phosphatase 1B; RAS: rat sarcoma; ERK1/2: extracellular signal-regulated kinase 1/2; PI3K: phosphoinositide 3-kinases; PDK1: phosphoinositide-dependent kinase-1; PP2A: protein phosphatase 2A; AKT: protein kinase B; GSK-3β: glycogen synthase kinase-3β; PPAR-γ: peroxisome proliferator-activated receptor-γ; AMPK: AMP-activated protein kinase.

The inhibition of intestinal glucose absorption is consistent with the observation of a reduction in glycaemia 1 h after glucose loading paralleled by a significant reduction of glucose absorbed in situ at the jejunum level by an aqueous extract of nettle (250 mg/kg by oral gavage). However, the same extract failed to reduce hyperglycemia in diabetic rats in which diabetes was induced by alloxan [177]. Specific inhibitory activity of the stinging nettle aqueous extracts on α-glucosidase and α-amylase enzymes was investigated and confirmed [95,178]. More recently, seco-isolariciresinol was identified as the phenolic compound responsible for the α-amylase and β-glucosidase inhibition [102]. Focusing on the increase of insulin secretion from the islets of Langerhans already reported in 2003 [179], compelling evidence comes from studies published between 2011–2021 and reporting the ability of stinging nettle to inhibit islet atrophy and promote regeneration of pancreatic β-cells. Indeed, recently, using the model of streptozotocin (STZ)-induced diabetes in rats, it was demonstrated that stinging nettle distillate (12.5 mL/kg/day for 4 weeks by oral gavage) was able to reduce hyperglycemia, increasing serum insulin levels, possibly correlated with the observed increase in β-cells number, an effect that was not shared by glibenclamide (0.6 mg/kg/day) or insulin (3 units/bid of protamine insulin) used as controls [99]. A positive effect of stinging nettle on pancreas proliferation was already observed in 2013 in STZ-induced diabetic rats: stinging nettle dried alcoholic and aqueous extracts induced an appropriate repair of pancreatic tissue [94]. Moreover, the administration of the stinging nettle aqueous extract to diabetic rats undergoing physical activity (swimming exercise) induced pancreatic β-cells proliferation, as demonstrated by hypercellularity revealed by histological analysis. These in vivo observations are in keeping with in vitro observations on RIN-5F pancreatic cells (an insulinoma cell line used as a β-cell model), which displayed a marked increase in glucose-stimulated insulin secretion with stinging nettle aqueous extract at both 1.5 mg/mL and 3 mg/mL [96]. Interestingly, the same study also provides evidence for the insulin sensitivity effect of stinging nettle. Indeed, rats treated with stinging nettle showed a significantly reduced HOMA-IR. Consistently, the extract stimulated in a concentration-dependent manner (1.5 mg/mL and 4 mg/mL) the glucose uptake by immortalized rat skeletal (L6) myoblast cells differentiated into a myotube muscle cell, phenotype naturally expressing GLUT4 [96]. GLUT4 translocation to the plasma membrane in both basal and insulin-stimulated state was demonstrated in L6-GLUT4myc cells, a rat muscle cells line, exposed to 125 and 250 μg/mL of nettle extracts [93]. Beneficial effects on glucose uptake were also observed in C2C12 myotubes. Stinging nettle extract at 5 μg/mL counteracted the AKT dephosphorylation induced by free fatty acids and has improved glycogen synthesis. The ex-vivo analysis of gastrocnemius muscle from obesity-induced insulin resistance mice confirmed the effect of stinging nettle on AKT phosphorylation [97], which could be related to decreased activity of cytosolic protein phosphatase 2A (PP2A) [98], a serine/threonine phosphatase activated by ceramides which affect insulin signaling [180,181] inactivating AKT [182]. The activation of the AKT pathway is known to deactivate the glycogen synthase kinase (GSK)-3β, a regulator of glycogen metabolism [183]. Consistently, the stinging nettle extract reduces blood GSK-3β levels, possibly through the activation of the Kirsten rat sarcoma (KRAS) pathway, which in turn activates several downstream signaling molecules, such as PI3K [100] and consequently AKT (Figure 2).

Apart from AKT phosphorylation, the extract (1–10 µg/mL) also improved the expression of adiponectin and the activation of ceramidases, thus decreasing ceramide accumulation, a predictor of T2DM development [184], in 3T3-L1 adipocytes exposed to free fatty acid [97]. Ex vivo experiments demonstrated in a model of high-fat diet (HFD) induced-insulin resistance that stinging nettle increased the expression of genes involved in adipogenesis (peroxisome proliferator-activated receptor (PPAR)-γ, CEBP-α and CD36) [101] and decreased the expression of all markers of lipogenesis and triglyceride synthesis (diacylglycerol O-acyltransferase 1 and fatty acid binding protein 4). In the liver, stinging nettle increases the expression of markers of fatty acid oxidation like PPAR-α, fork-head box (FOX)O1 and carnitine palmitoyl-transferase (CPT)-1a [101]. All these described molecular effects were paralleled by a reduction in weight gain, fasting plasma glucose, insulin level and the HOMA-IR or glucose tolerance, both when stinging nettle supplementation was used as a preventive approach (supplementation started at the beginning of the study) [98] and when used as a treatment approach (after 6 weeks of HFD) [101]. Similar results were obtained administering an hydroalcoholic extract of stinging nettle to fructose-induced insulin resistance rats [91].

Another exciting approach pursued in evaluating the anti-diabetic effect of stinging nettle deals with the ability of its extract to counteract the different complications associated with diabetes, from memory functions affected by alteration in the muscarinic cholinergic system in the hippocampus [95] to depressive behavior and cognitive dysfunction after dexamethasone-induced hyperglycemia [185], deterioration of seminiferous tubules [186] and even diabetic nephropathy [187]. This latter effect was investigated in vivo with the model of STZ-induced diabetic nephropathy in STZ-diabetic mice, a well-known and reproducible model of diabetes in which renal injury shares similarities to human diabetic nephropathy [188]. The combination of stinging nettle hydroalcoholic extract with pioglitazone showed a synergic effect on the reduction of blood glucose and prevention of renal dysfunction, measured in terms of serum urea and creatinine. The mechanism suggested for these effects was related to the anti-oxidant properties of the plant: stinging nettle significantly reduced reactive oxygen species (ROS) formation in renal supernatant, levels of malondialdehyde (MDA) and protein carbonyl and oxidation of glutathione (GSH) [187]. Interestingly, the stinging nettle extract administered to STZ rats attenuated the diabetes-related cardiac dysfunction and improved the mitochondrial function in the cardiac muscle by restoring NRF2 and the peroxisome proliferator-activated receptor-gamma coactivator (PGC)-1α deficits. These results suggested that stinging nettle could have a protective effect on cardiac tissue, thus minimizing the incidence of diabetic cardiomyopathy [189].

### 4.2. Yarrow

Well-characterized both in vitro and in vivo is also yarrow, for which different molecular pathways were investigated. Yarrow, thanks to its multiple active ingredients, ranging from monoterpenes and sesquiterpenes and phenolic compounds [67,68,69,70,71,72] to alkaloids, [73,74] showed different mechanisms of action that can contribute to the anti-diabetic effect demonstrated in several in vivo studies. The first common effect was β-cells protection, possibly associated with a reduction of inflammatory mediator release. Using the T1DM model of alloxan-induced diabetes in rats, it has been demonstrated that both an aqueous and a methanolic extract of yarrow at 500 mg/kg i.p. partially restored the normal cellular population and enlarged size of diabetic β-cells, with comparable effects despite the different yield of the two extracts (2.6% and 7.3% for aqueous and methanolic extracts, respectively) [103]. Interestingly, the effects shown after 14 days of treatment were comparable to those of glibenclamide (30 mg/kg i.p.). The protective effect on the pancreas was paralleled by the significant increase in glucose tolerance and reduced fasting glycemia, where the methanolic extract seemed to be the most effective [103]. These beneficial effects could be related to the reduction of interleukin (IL)-1β and inducible nitric oxide synthase (iNOS) mRNA elicited by yarrow [77]. Indeed, both IL-1β and iNOS can be considered effectors of β-cell destruction in T1DM, which are thought to act as early inflammatory signals [190]. A hydroalcoholic extract (80% of ethanol) of aerial parts of yarrow 100 mg/kg/day i.p. for 14 days caused a statistically significant reduction of STZ-induced hyperglycemia, with insulin serum levels restored to negative controls [77]. The authors suggest that the effects could be due to the protection of β-cells promoted by the downregulation of IL-1β and iNOS. Moreover, the pancreatic protection could be due also to the over-expression of PPARγ. Indeed, PPARγ activation has been reported to decrease the expression of inflammatory mediators, such as IL-1β and iNOS [191]. The hypothesis of PPARγ contribution comes from the observation that the hydroalcoholic extract induces the over-expression of PPARγ and that of the glucose transporter GLUT4 (Figure 2) in the preadipocytes cell line 3T3-L1. These events could drive the beneficial effect registered for insulin sensitivity in vivo, where this extract at 100 mg/kg i.p. was able to improve the oral glucose and sucrose tolerance tests in a model of chemically induced T2DM (nicotinamide 20 mg/kg i.p. followed by STZ 120 mg/kg i.p.) in CD1 mice [105]. These data highlight the potential insulin-sensitizing action of this plant. Simultaneously, using RINm5F cells, the same authors demonstrated that the hydroethanolic (70% of ethanol) extract of aerial parts of yarrow at 200 µg/mL induced an increase in intracellular Ca^2+^ concentration and accordingly of insulin secretion comparable to that of glibenclamide [105]. Consistently, the same study demonstrated, using the OGTT, that yarrow elicits a hypoglycemic response in normoglycemic CD1 mice, thus supporting that it could act as an insulin secretagogue. However, the possibility of a direct effect on the α-glucosidases was also evaluated in vitro on homogenate of Sprague Dawley rats’ intestinal brush border: the yarrow extract inhibited the α-glucosidases activity by 55% [105], confirming previous data [104]. However, the anti-diabetic effects of yarrow could also be ascribed to a more general anti-oxidant potential of the plant observed when exploring the protective effect of a hydroalcoholic extract of yarrow (300 g of plant in 70% ethanol) on lipid profile, blood glucose levels, body weight and serum liver enzymes in a model of T1DM, STZ-induced, in rats [192]. The oral gavage with the yarrow at 50 mg/kg/day or 100 mg/kg/day for 28 days increased the body weight of both non-diabetic and diabetic rats [192]. Similar effects were already reported [103]. These data should be interpreted according to the model used: STZ-model causes a significant reduction in the body weight of diabetic animals, one of the typical T1DM features [193,194,195]. In the same model, yarrow supplementation at 100 mg/kg/day reduced blood glucose levels to a similar extent of metformin at 250mg/kg/day [192]. Comparable results were obtained in an HFD-induced insulin resistance model using a CO_2_ supercritical fluid extract from yarrow. The extract at 800 mg/Kg, five times per week for 3 months, improved fasting glycaemia, oral glucose tolerance, insulin levels and HOMA-IR [196]. The beneficial effects on hyperglycemia and hepatic enzymes were paralleled by ameliorating lipid profile [103,192,196,197,198]. These effects were, at least in part, ascribed by the authors to the inhibition of fatty acids synthesis. However, the plant’s accurate and complete mechanism of action still needs to be fully elucidated. 

### 4.3. Bilberry

There is much evidence of the anti-diabetic properties of plants rich in anthocyanins, such as bilberry, blackberry and raspberry.

The use of bilberry leaf for diabetes was widespread at the beginning of the 20th century, before the discovery of insulin, was one of the most frequently used herbal anti-diabetic remedies [110]. Many bilberry leaf extracts are available on the market and bilberry leaf tea is still recommended and sold by more than 40% of herbalists to manage diabetes [110]. Its efficacy has been confirmed in vivo [108]. In this study, the dietary supplementation with bilberry fruit powder (2 g/day by oral gavage) for 4 weeks significantly reduced glycemia in diabetic rats. The effect on hyperglycemia was comparable to that of glibenclamide at 0.6 mg/kg i.p. Still, bilberry was also effective in improving the lipid profile [108]. Similar beneficial effects were observed for the bilberry leaf extract (2% for 50 days) in a model of diabetes induced by STZ in combination with a high-carbohydrate diet in Wistar rats with diabetes [199]. The effect could be due to the anthocyanin components, as suggested by using the model of Zucker fatty rats (ZDF) [200], which spontaneously develop obesity and insulin resistance, progressing into hyperglycemia with impaired β-cell function and decreased responsiveness to insulin and glucose [201]. In the same experimental model, bilberry was demonstrated to be effective only on the total and LDL-cholesterol levels, not on the glycemic profile [106]. These contrasting results could be due to the experimental protocol adopted with bilberry administered as a 15% substitute for the standard diet [106], or as a non-acilated anthocyanin extract from bilberry at 25–50 mg/kg/day [200].

Notably, the beneficial effects of bilberry on diabetes could be far beyond the anti-hyperglycemic: the supplementation with bilberry extract (100 mg/kg) orally administered to STZ-induced diabetic rats for 6 weeks could decrease markers of diabetic retinopathy, such as retinal vascular endothelial growth factor expression, even though it did not affect blood glucose levels and body weight [107]. The data reported is in keeping with a protective effect on retina [202]. The retina protection observed is not surprising for bilberry. At the same time, pilot data on the beneficial effect on hippocampal neurons/nerve fibers plasticity [203], if confirmed, could represent an important milestone in determining the functional role of bilberry supplementation in diabetes. On the contrary, using a model of chemically induced T2DM (the STZ+nicotinamide non-insulin-dependent model), no protective effects were observed on diabetic gastroenteropathy [204]. 

The direct anti-diabetic activity could be correlated to the ability of bilberry extracts to inhibit the activity of carbohydrate-hydrolyzing enzymes such as α-glucosidase and α-amylase, thus reducing postprandial hyperglycemia. This effect, which may be related to flavonoids [112], was demonstrated in different studies. The inhibitory activity of α-glucosidase of phenol-rich extracts obtained from bilberry fruits from different Bulgarian mountains have been tested [111]. The comparative evaluation of water (total polyphenolic content in extracts expressed as gallic acid equivalents, gallic acid equivalent (GAE)/100 mg, ranging from 290.83 ± 8.72 to 453.63 ± 17.23), acetone (GAE/100 mg ranging from 510.88 ± 16.10 to 636.46 ± 22.90) and methanol (GAE/100 mg, ranging from 548.30 ± 16.75 to 1299.60 ± 32.46) extract highlighted that the methanol extract was the most efficient in inhibiting the α-glucosidase activity, probably because of the highest amount in gallic acid, in accord with a previous report [169]. Leaf extracts showed good anti-oxidant power, too and the hydroethanolic one significantly inhibited α-glucosidase activity, with an IC_50_ value equal to that of acarbose [110]. On the other hand, the activity of leaf extract on α-amylase was not straightforward. Both aqueous and hydroethanolic extracts were unable to inhibit α-amylase activity [110]. However, bilberry fruits showed a positive response in previous screening [109]. In addition, recent evidence demonstrated that methanol or aqueous extract of bilberry fruits could inhibit 80% of the glucose uptake in Caco-2 cells [113].

The anthocyanin component, more than the flavonoid or phenolic, contributes to the beneficial effects of bilberry, with its potent anti-oxidant activity. Indeed, a comparative study on the anti-oxidant activity of hydroethanolic (75% of ethanol) extracts from blueberries, bilberries, cranberries and mulberries [112] demonstrated that the anti-oxidant activity follows the anthocyanin content: blueberry > bilberry > mulberry > cranberry. A parallel hierarchy was also found for the inhibitory effect on protein tyrosine phosphatase 1B (PTP1B), a negative regulator of the leptin and insulin signaling pathways [205] involved in insulin resistance [206]. Therefore, it is possible to speculate that bilberry could improve glucose homeostasis by inhibiting PTP1B (Figure 2), as muscle-specific PTP1B^−/−^ mice exhibit improved muscle glucose uptake, systemic insulin sensitivity and glucose tolerance [207]. On the contrary, mulberry was the most effective in α-glucosidase activity [112]. 

### 4.4. Raspberry

Apart from bilberry, anthocyanins are particularly abundant in plants belonging to the Rosaceae Family, *Rubus* genus. Looking at raspberry, the anthocyanins are responsible for the berry’s brilliant red color (also known as red raspberry). Red raspberry is rich in dietary fiber (6 g in 1 cup of frozen whole fruit) that may provide glycemic control benefits [208]. However, the plant also contains different micronutrients, such as folic acid and vitamins C and K, magnesium, potassium, calcium and iron. Still, it is poor in carbohydrates and consistently has a low glycemic index that supports its possible health-promoting benefits in diabetes. Alongside anthocyanins, other polyphenols, such as flavanols, flavonols and phenolic acid, such as ellagitannins [209], are the main bioactive compounds found in red raspberry. Altogether, these active ingredients may participate in the anti-diabetic effect of red raspberry. The proposed mechanism for the modulation of postprandial glycemia following consumption of dietary berries or their extracts is the inhibition of the enzymes α-amylase, even higher than realized by other berries, and α-glucosidase to prevent glucose absorption in the intestines [115,118]. Similar effects were registered in vitro for another species of the *Rubus* genus, *Rubus grandifolius* Lowe, an endemic species from the Madeira archipelago, whose leaf (composed of 44–49% of ellagitannins, followed by hydroxycinnamic acids, flavonols and flavones) and berry (anthocyanins 54%, ellagitannins 37–39%, flavonols 8–9% and hydroxycinnamic acids 0.7–0.9%) extracts strongly inhibited yeast α-glucosidase, showing lower IC_50_ values than acarbose, with leaves being the most active samples [210]. The microbial metabolite of ellagitannins/ellagic acid, urolithins, were shown to play a role in glucose homeostasis [211]. However, anthocyanins are particularly involved in the beneficial effects of red raspberry and other *Rubus* species, including improved insulin response, glucose and lipid metabolism, and anti-oxidant and anti-inflammatory properties. Anthocyanins may enhance insulin secretion from pancreatic β-cells, thus improving insulin sensitivity [110]. A key role in these events is ascribed to AMPK, highlighted using knockout mice for the catalytic subunit of AMPK (AMPKα1^−/−^ mice) [116]. An HFD supplemented freeze-dried raspberry (5% of dry fruits, containing polyphenols at 11 g gallic acid equivalent/kg of dry weight) for 10 weeks had beneficial effects on body weight and expression of pro-inflammatory cytokines, such as TNF-α, IL-1β, IL-6 and IL-18, only in wild-type animals and not in the AMPKα1^−/−^ counterpart. Moreover, raspberry supplementation reduced lipid accumulation in skeletal muscle and increased GLUT4 mRNA and proteins (Figure 2). These effects were observed only in wild-type mice, in which raspberry induced a significant increase of phospho/total-AKT ratio [116], a well-known downstream effector of insulin signaling. Raspberry supplementation (5% freeze-dried fruits for 12 weeks) in HFD C57BL/6J obese mice activates the AMPK/Sirt1 pathway and increases the FNDC5/irisin contents, thus decreasing white adipose tissue hypertrophy, suppressing pro-inflammatory cytokines expression and macrophage infiltration in white adipose and improving insulin sensitivity [117]. Consistently, using a similar animal model, the consumption of the equivalent of a single daily serving of either raspberry puree concentrate or raspberry juice concentrate for 10 weeks in C57BL/6J mice fed an HFD was demonstrated to ameliorate the symptoms of metabolic syndrome, by inducing, among other effects, a significant reduction of glucose and insulin levels in comparison to HFD animals [212]. Interestingly, the preventive supplementation of raspberry improved insulin resistance and metabolic dysfunction in diet-induced obesity. These effects were associated with the suppression of the nucleotide-binding oligomerization domain, leucine-rich repeat and pyrin domain-containing (NLRP)3 inflammasome elicited by HFD [114]. The ability of raspberry to counteract diet-induced obesity and metabolic dysfunction, particularly to inhibit NLRP3 inflammasome, was investigated by comparing the effects of polyphenol fractions from pulp, whole fruit and seed [213]. Their results suggested that supplementation with raspberry polyphenols from pulp and, to a lesser extent, from entire fruit effectively prevents HFD-induced weight gain, insulin resistance and energy expenditure. The data is consistent with the inhibitory effect on NLRP3 in adipose tissue macrophages of epididymal white adipose tissue, which was higher with the pulp polyphenol fraction and less extensive for polyphenols from whole fruits. Notably, anthocyanins are the primary constituents of pulp polyphenols, while the seed fraction contains the highest amount of ellagic acid and epicatechin. Unexpectedly, the supplementation with seed polyphenol fractions failed to exert metabolic benefits. However, these results highlight anthocyanins’ properties and crucial role in this extract [213].

### 4.5. Blackberry

Similar protective effects against hyperglycemia from anthocyanins were also recorded for blackberry. The extracts from its berries were tested as anti-hyperglycemic agents on diabetic rats (using the T1DM model by STZ) after anthocyanins extraction with 80% acidified ethanol in ultrasonic conditions at room temperature [214]. The extract (administered for 5 weeks in drinking water to obtain a concentration expressed as cyanidin-3-glucoside of 300 mg/L) determined a significant decrease in glucose level from 360 to about 270 mg/dL [214]. Previous findings suggested that the hypoglycemic effect of the blackberry is independent of the elevation of insulin secretion but is likely mediated by extra-pancreatic actions, such as the increase of glucose uptake from peripheral tissues [215]. This hypothesis was supported by the results obtained administering a *Rubus* extract (blackberry Loch Ness variety, obtained by lyophilization and extraction with 80% methanol in 0.1% HCl) in rats fed a standard diet or cafeteria diet [119], a hypercaloric diet model based on the recapitulation of oro-sensory properties and palatability of food promoting its overconsumption [216]. Interestingly, the authors showed that blackberry extract, in rats given a standard diet, decreased glycemia and increased insulin sensitivity, as demonstrated by the reduction in the HOMA-IR, with a sex-dependent response greater in females than in males. In parallel, animals showed decreased plasma glucose and insulin and increased plasma non-esterified fatty acids, glycerol and 3-hydroxybutyrate and triacylglycerols concentrations. However, no effect was seen in rats fed the cafeteria diet, probably due to their high insulin resistance [119].

In parallel, blackberry Schott variety was tested in vitro on the human hepatoma cells line HepG2. The glucose consumption in HepG2 cells was increased in a concentration-dependent manner by the different blackberry extracts: blackberry ethanol extract, gastrointestinal digested blackberry and gut metabolites of blackberry collected at 24 and 48 h fermentation. A similar significant anti-oxidant activity, most probably due to the phenolic metabolites, was observed in cells exposed to oxidative stress induced with high glucose (30 mM) plus palmitic acid (0.2 mM) for 24 h. Indeed, all the extracts decreased the ROS content and restored the cellular GSH and mitochondrial membrane potential [46].

### 4.6. Wild Strawberry

Still belonging to Rosaceae, a plant rich in anthocyanins is wild strawberry, which also contains high amounts of vitamin C, folate and phenolic constituents [5]. The anti-tumor, anti-oxidant and anti-diabetic properties of its fruits have been investigated. Standard mechanisms are based on their anti-oxidant and anti-inflammatory properties. A hydroalcoholic extract from wild strawberry (160 µg/mL) exerted on the macrophage cell line Raw 264.7 a direct nitric oxide scavenging activity. Moreover, the proteasome activity was inhibited and the conversion of the microtubule-associated protein light chain LC3-I to LC3-II, suggesting autophagy, was promoted [51]. The literature on the diabetic effect of strawberries is based on different plants belonging to the *Fragaria* genus. For instance, one of the most studied is *Fragaria* x *ananassa*, which differs from *Fragaria vesca* L. for the composition of volatile esters (acetate in *Fragaria vesca* L. and ethyl hexanoate in *Fragaria* x *ananassa*) [217,218]. The freeze-dried strawberry powder obtained from *Fragaria* x *ananassa* was tested as a supplement (700 mg/kg/day for 45 days) in diabetic rats (T1DM according to the alloxan model) [219]. The extract reverted hyperglycemia and insulin levels to near normal in that study. 

Although the mechanism underlying these effects remains to be completely clarified, evaluating the strawberry extract’s ability to inhibit metabolic syndrome-related enzymes in vitro showed positive results. In particular, the extract inhibited the α-glucosidase and α-amylase enzymes, thus supporting that this mechanism could underlie, at least in part, its anti-diabetic effect [118,120]. However, another study failed to demonstrate any decrease in hyperglycemia in a model of HFD rats following supplementation with wild strawberry fruit ethanolic extract up to 500 mg/kg/day for 30 days [220]. 

### 4.7. Elderberry

Elderberry represents again a plant with fascinating anti-diabetic properties, whose mechanism has been previously compared to that of sulfonylureas, having similar insulin-releasing action counteracted by diazoxide [221]. The beneficial effects of elderberry are related to phenols and anthocyanins, whose amount in fruits is even higher than in other plants [61]. Notably, these active principles are heat-stable, acetone-insoluble and unaffected by altered pH environment [222]. Apart from promoting insulin secretion from pancreatic cells, the aqueous extract of elderberry has insulin-sensitizing properties. It is well characterized in the muscles, where it can directly stimulate glucose uptake and glucose metabolism [222,223]. These observations have been confirmed in in vitro study on pig primary myotube cells [122]. The authors showed that the dichloromethane and the methanol elderflower extracts are both dose-dependent (6−1000 μg/mL) and able to increase glucose uptake, with the dichloromethane extract being more effective. Similarly, it was demonstrated that the 96% ethanol and the acidic methanol extracts of elderberry at 50 μg/mL increase glucose uptake in human skeletal muscle cells by 37.4 ± 4.8 and 42.6 ± 6.7%, respectively. Notably, both extracts were strong α-glucosidase and α-amylase inhibitors, maybe to their high content of polyphenols. The further evaluation of the anti-oxidant activity of these extracts evidenced that the acidic methanolic extract (IC_50_ 95.9 ± 3.9 μg/mL) showed the highest radical scavenging activity, followed by pressed juice and the 96% ethanol extract (IC_50_ 128.3 ± 4.3 μg/mL) [125]. The same authors achieved similar results on HepG2 cells [126]. Muscle and liver are not exclusive targets of the elderberry, which also affects adipocytes. demonstrated that elderberry extracts increases glucose uptake in mature, fully differentiated insulin-resistant 3T3-L1 adipocytes more efficiently than rosiglitazone. In parallel, the authors observed a reduction in ROS and an improvement in adipokine balance: leptin is down-regulated and adiponectin is up-regulated [127]. This could promote a non-obese phenotype, supporting the observation of a reduction of fat accumulation in Caenorhabditis elegans treated with elderflower extract [122]. Together, these observations strengthen the hypothesis that this plant could exert beneficial effects on glucose utilization. Nevertheless, according to the above mentioned results [122], the possible induction of cellular stress as the mainstream mechanism of elderberry extracts on glucose uptake can be discharged. Evaluating the effects of elderflower extracts on the transcription regulation of heme oxygenase 1 (HMOX1, a marker of oxidative stress response) and heat shock protein 70 (HSP70, whose expression is induced by different kinds of cellular stress conditions), no indication of cellular stress on the myotubes were observed. In contrast, dichloromethane extract showed significant anti-oxidative properties by reducing the amount of intracellular ROS. Interestingly, PPAR-γ has been implicated in the insulin sensitization effects exerted by elderberry: the extract activated PPAR-γ (Figure 2) in COS-1 cells (fibroblast-like cells isolated from the kidney of an African green monkey), probably because of the presence of α-linolenic acid, linoleic acid and polyphenols such as naringenin [121]. However, in vivo contrasting results were reported. Female db/db-mice supplemented for 4 weeks with a methanol extract of elderflowers (0.1 and 1 g/kg/die), placebo, or rosiglitazone (0.02 g/kg/die) had no benefit from elderflower extract in terms of fasting plasma glucose, adiponectin and cholesterol levels [121]. 

On the contrary, the supplementation for 4 weeks with a lipophilic and polar extract (190 and 350 mg/kg/die, respectively) decreased fasting blood glucose, modulated insulin secretion and improved insulin resistance, as demonstrated by the HOMA-IR index in Wistar STZ-induced diabetic rats fed with an HFD. The comparison between the two extracts indicated the polar one as the most active on fasting blood glucose and the lipophilic on plasma insulin levels [124]. Similar results were achieved in HFD-fed mice supplemented with an anthocyanin-rich black elderberry extract displaying a reduction in fasting insulin, HOMA-IR index and fasting serum triacylglycerol. Systemic inflammation was attenuated as serum MCP-1 and TNFα were reduced by 30% [123,224]. 

Some observations suggest a potential role for black elderberry also in diabetes complications. Acute and sub-chronic in vivo effects of elderberry were evaluated in an alloxan-induced diabetic mouse model of neuropathic pain. The study confirms elderberry’s hypo-glycemic effect: the extract, containing the most active fraction of quercetin, kaempferol and isorhamnetin, reduced the blood glucose level both acutely (after 6 h) and chronically (on the 8th day of treatment). Moreover, a significant increase in body weight (suggesting an improvement of the catabolic deterioration associated with the alloxan-diabetes model mimicking the T1DM) was registered. Interestingly the elderberry extract at 50–200 mg/kg acutely improved the tactile allodynia and chronically ameliorated thermal hyperalgesia in a dose-dependent manner [225]. In the same year, the effects on immune system imbalance of a dried and powdered fruit extract obtained using acidulated methanol were studied in Wistar white male rats with diabetes induced by the injection of a single dose of STZ [226]. Elderberry extract reduced TNF-α and interferon (IFN)-γ levels in diabetic animals. It limited the production of fibrinogen, thus evidencing an anti-inflammatory effect helpful in reducing the development of microvascular diabetic complications. Consistently, in activated RAW 264.7 macrophages, the extract of elderberry down-regulated the expression of proinflammatory genes such as TNF-α, IL-6, COX-2 and iNOS [127]. Altogether, these findings suggested a potential role of elderberry in reducing insulin resistance, lipo-toxicity and inflammation, with a possibly beneficial effect on both prevention of diabetes and its complications.

### 4.8. Rosehip

Used as a folk remedy in treating diabetes in Turkey [227], rosehip has been extensively studied for its anti-inflammatory, anti-obesity and anti-diabetic activity [57,228,229]. Two primary mechanisms are proposed for the anti-diabetic properties: the inhibition of α-amylase enzymes and the stimulation of insulin secretion by protecting pancreas damage. The first mechanism was pointed out evaluating the anti-oxidant activity and α-amylase inhibition of a methanolic extract (1:10; total phenolic content expressed in GAE = 21.918 mg/g and complete flavonoids as rutin equivalents (RE) = 2.65 mg/g) of pseudo-fruit and flowers from rosehip [130]. Results of the preliminary agar diffusion of amylase inhibition assays indicate that rosehip extracts exhibit a complete inhibition of α-amylase enzyme; that was confirmed by the α-amylase inhibition quantitative assay, with a 100% inhibition for extract concentration of 5.5 mg/mL. This effect was ascribed to vitamin C, one of the active ingredients of rosehip alongside tocopherols, carotenoids, phenolic, organic acids, sugars and essential fatty acids [57]. Consistently, a significant free radical scavenging activity was observed [130].

Both in vitro and in vivo studies demonstrated the protective effect of rosehip on β-cells. An aqueous extract of ripe fruits at 0.001 mg/mL significantly enhanced cell number and morphological alterations in pancreatic βTC6 cells, acting as a growth factor for the pancreatic β-cell line [129]. This could contribute to the beneficial effect of rosehip in diabetes, although no significant protective effect was observed after STZ cell exposure. However, STZ cytotoxicity mimics alterations in T1DM, but the proliferative effect observed on βTC6 cells suggests that this plant could be effective when β-cells are still at least partially preserved, as in at least the early phases of T2DM. Moreover, the amount of active ingredients, especially the oligosaccharide fraction, could account for that. Indeed, in vivo demonstrations again favor a beneficial effect on the pancreas in models of STZ-induced diabetes. The daily oral administration of a hydroalcoholic extract (70% ethanol) of rosehip (5.9% ± 0.4% of gallic acid equivalent/100 g fraction) at 250 mg/kg or 500 mg/kg for 6 weeks was effective as glibenclamide (600 µg/kg) in reducing fasting blood glucose levels, improving in the pancreas the necrotic islets and increasing the number of islets [128]. In parallel, a reduction in serum triglycerides has been observed. This data is consistent with the findings that treating the hydroalcoholic extract of rosehip improves liver histology and liver enzyme activity compared to untreated diabetic rats [230]. Interestingly, rosehip extract showed positive effects if administered to animals with pre-diabetic status. In particular, it was demonstrated that spontaneously diabetic Torii rats supplemented with rosehip extract at 100 mg/kg body weight for 12 weeks preserved pancreatic β-cell function, promoted insulin secretion, improved glucose tolerance and suppressed advanced plasma glycation end-products formation [131]. These effects are probably due to the oligosaccharide fraction in rosehip extract. Indeed, the authors purified and characterized this fraction and demonstrated that the supplementation with isolate oligosaccharide (8–40 mg/kg bid) for 21 days to STZ-diabetic rats increased β-cell mass, suggesting the regenerative potential of the oligosaccharide fraction from rosehip [133]. An analysis of the expression of genes involved in the function and proliferation of β-cells showed that the extract also increases *Ngn3*, *Nkx6.1* and *Ins* expression. Moreover, this supplementation decreased blood glucose levels, gluconeogenesis and α-glucosidase activity and improved the OGTT test. Similarly, the purified oligosaccharide (2 mg/kg) was even more effective than the crude extract of rosehip (40%) in decreasing fasting blood glucose in diabetic rats. Oligosaccharides also increased blood insulin levels and the number and size of pancreatic islets, indicating an increase in the number of β-cells [132]. 

Moreover, oligosaccharide was able to increase insulin secretion similarly to glibenclamide. Molecular analysis revealed that oligosaccharides increased the expression of *Ins1* and pancreatic and duodenal homeobox 1 (*Pdx1*) insulin-producing genes, which led to an increase in the expression of insulin-dependent genes such as *Gck* and *Ptp1b*. On the other hand, the expression of the solute carrier family 2 member 2 (*Slc2a2*) gene is related to the GLUT-2 transporter [231], was significantly reduced by increased insulin concentrations. Finally, the same authors deepened the molecular mechanism of the anti-diabetic effect of the oligosaccharide fraction from rosehip, focusing on its effect on DNA methylation, whose alteration has been reported in diabetic humans and animal models [128]. Diabetic rats receiving isolated oligosaccharides (10, 20, or 30 mg/kg) twice daily for 4 and 8 weeks showed a significant increase in body weight and a decrease in blood sugar level compared to their untreated counterpart. In parallel, treated groups displayed reduced DNA methylation, as demonstrated by the decreased expression of DNA methyltransferases (DNMT) such as *Dnmt1* and *Dnmt3α* in both pancreas and the blood. On the other side, Dnmt3β expression was reduced in the pancreas and increased in the blood, suggesting that oligosaccharides may interact with some upstream signaling factors of DNMT. Furthermore, the increased expression levels of the *Pdx1*, *PTP1B2*, *Ins1* and *Gck* genes observed in the oligosaccharide-treated group may be related to the hypomethylation effect of this fraction in diabetic rats [232]. Interestingly, the evidence against a possible role of rosehip on the metabolism of glucose by hepatic cells was provided. Indeed, the extract did not cause a significant glucose-lowering effect in the human hepatocellular carcinoma cell line exposed to hyperglycemia (11.1 mmol/L) [129]. On the contrary, an additional mechanism of action for rosehip oligosaccharides could be related to the modulation of the autophagic pathway. The activation of autophagy has been associated with the downregulation of the microRNAs (short non-coding RNAs involved in silencing RNA targets to regulate gene expression and cellular function) miR-21 and miR-22, in particular [233,234]. These two microRNAs have also been implicated in diabetes mellitus [235]. Using Rin-5F, a secondary clone of the rat islet tumor cell line RIN-m used as a model of pancreatic β-cells, the oligosaccharide fraction from rosehip was demonstrated to increase the expression of autophagy markers, such as miR-21, miR-22, AKT (Figure 2), autophagy-related 5 (ATG5), Beclin1, LC3A and LC3B, suggesting that rosehip anti-diabetic effects are mediated at least in part by the modulation of the autophagy pathway [236]. 

The anti-diabetic effects of cornelian cherry fruits seem to be related to their significant amounts of bioflavonoids (anthocyanins), ursolic acid and vitamin C, in particular [152,237,238,239,240]. Anthocyanins have been recognized to exert anti-diabetic effects increasing insulin sensitivity, protecting hepatic function and exercising a general anti-oxidant effect. 

### 4.9. Cornelian Cherry

The cornelian cherry ability to reduce hyperglycemia is well documented and consistent between different models such as the alloxan model [241], the STZ model [135] and the T2DM model of ZDF [134]. However, the increase in insulin sensitivity by cornelian cherry is under debate. The effects of red and yellow fruit extracts [20 mg/kg/day (mean of total polyphenols content of about 350 GAE/g and mean anti-oxidant activity of about 2.70 Trolox equivalent/g), by oral gavage for 14 days] was evaluated in STZ-induced diabetes rats. However, the same authors did not confirm these positive results when administered a pure loganic acid extract from cornelian cherry being this the main iridoid glycoside in the cornelian cherry fruits [135]. The authors specified in the discussion that it is possible that the 20 mg/kg/day for 14 days dosage of pure loganic acid extract could be too low to be effective. Similar effect on hyperglycemia, but not on HbAc1 or insulin sensitivity were reported by Capcarova et al. [134]. However, insulin sensitivity was measured in terms of HOMA-IR instead of OGTT. Moreover, it is essential to underline that the study from Dzydzan et al. [135] was focused on the T1DM model, while that from Capcarova et al. [134] used the T2DM model of ZDF rat. Also, time and dosages were different: 20 mg/kg/day for 14 days [135] versus 1000 mg/kg/day for 10 weeks [134], respectively. Another investigated mechanism underlying the anti-hyperglycemic effect of cornelian cherry extract is the ability to inhibit the α-glucosidase and α-amylase enzymes. Loganic acid, cornuside, cyanidin 3-galactoside and pelargonidin 3-glucoside were identified as the most dominant compounds, presenting ≥ 90% of all detected iridoid and phenolic constituents in the extracts and pointed out pelargonidin 3-robinobiosby analyzing ide as the most potent inhibitor of α-glucosidase [137]. A 2 g/day homogenous powder of cornelian cherry for 4 weeks was also ascribed to liver beneficial effect, reducing hepatic inflammation in diabetic Wistar rats [241]. This anti-inflammatory effect could be related to the anti-oxidant property of the plant extracts. They induced an increase of GSH and thio-barbituric acid reactive substances (TBARS), a decrease in protein carbonyl groups and advanced oxidation protein product levels, all markers of oxidative stress and subsequent damage to proteins [135]. The loganic acid extract, in particular, increases the GSH content, GPx and glutathione reductase in leukocytes, thus reducing ROS production [136].

## 5. The Anti-Diabetic Effects of Plants from the Occitan Valleys of the Piedmont Alps: Clinical Evidence of Efficacy

The clinical studies demonstrating the anti-diabetic efficacy of Alpine plants are scant and sparse. As expected, the Alpine extracts were tested in T2DM patients as supplements. Consistently, the tested common hypothesis is that these plants could be effective as an add-on-therapy of T2DM in a combinatorial approach with traditional anti-diabetic drugs to improve their efficacy.

As observed for experimental studies, the number of trials published between 2011 and 2021 is highly variable, with no analysis, to the best of our knowledge, on the use of elderberry in diabetic patients. Similarly, in 2011 the lack of human trials on yarrow was pointed out [65]. Nowadays, to the best of our knowledge, on the clinicaltrials.gov register, only one study aims to evaluate the use of this plant in diabetic patients. The study, completed in February 2019 (ClinicalTrials.gov Identifier: NCT04054284), seeks to assess the safety and efficacy of an herbal tea mixture in T2DM, including yarrow among the components of the mix without anti-diabetic properties (control treatment) in comparison with a combination with anti-diabetic properties and including stinging nettle and bilberry. The data has not been published yet, but the use of yarrow as a plant without anti-diabetic properties should be carefully interpreted, considering the type of preparation (herbal tea), the exact dosage and the active ingredient titration.

### 5.1. Stinging Nettle

The number of clinical trials on stinging nettle underlines the interest in the therapeutic potential of this plant. As reported by a recent meta-analysis, the effect on fasting glycaemia in T2DM is apparent [242]. It is reasonable to expect similar results from the ongoing study NCT00422357, investigating the hypoglycemic effects of stinging nettle tea bags in T2DM patients. On the contrary, no significant reduction was observed in insulin levels, HOMA-IR index, or HbA1c percentage [242]. Conflicting results have been reported from the different trials included. As written by the authors, the discrepancy is almost on biochemical outcomes, which are favorable for some [243,244,245,246,247,248] and unaffected for others [249,250]. For instance, it was observed that adding to the standard anti-diabetic therapy (mainly metformin or glibenclamide) hydroalcoholic extract of stinging nettle (45% ethanol, 55% water and 2.7 g of dry plant in 1 L extract) 100 mg/kg in 3 portions a day (after each main meals) for 8 weeks, was not superior to placebo in affecting insulin concentration and insulin sensitivity in patients of both genders (29.2% man and 49.3% women) with T2DM (mean duration of diabetes at baseline more than 8 years, HbAc1 ≤ 10%) [251]. In parallel, no differences were registered in circulating TNF-α, but the extract significantly reduced serum IL-6 and high-sensitivity C-reactive protein (hs-CRP) [251]. However, the clinical results confirm the anti-oxidant activity of the extract that increased the total anti-oxidant capacity and SOD, although no significant differences were registered for MDA and GPx [252]. Similar results on SOD levels were also reported in a third randomized double-blinded clinical trial involving just women over 50 years old affected by T2DM (mean duration of diabetes 13 years, HbA1c ≤ 10%) who received, as add-on therapy, placebo or the hydro-alcoholic extract of stinging nettle 100 mg/kg in 3 portions a day for 8 weeks [253]. Nitroxide levels were also increased in the treated group, suggesting that the extract could prevent the development of cardiovascular complications [254], as indicated by in vivo data cited above [187]. 

### 5.2. Wild Strawberry

Another plant widely studied in clinics is the wild strawberry. Notably, in the period included in the present review (2011–2021), very few experimental and preclinical papers were found in the PubMed database, while a plethora of studies demonstrated that consuming strawberries attenuates unfavorable postprandial responses or reduces the cardiovascular risk in subjects with metabolic syndrome clinical trials [255,256,257,258]. Moreover, a search on ClinicalTrial.gov in June 2022 retrieved five studies for wild strawberry as active treatment and diabetes: the NCT05362968, just approved and not yet recruiting; the NCT02610179, recruiting; and the completed studies NCT01199848 [259,260], NCT02607007 and NCT01766570 (no results available for both). This could reflect a higher stage of development of a strawberry-based approach as an add-on therapy for diabetes compared to other Alpine plants. For instance, a randomized, single-blind, placebo-controlled, cross-over trial demonstrated in overweight adults that the aqueous acetone strawberry extract (70%) containing 10 g of freeze-dried powder 81.6 mg/of anthocyanins (54% pelargonidin-3-O-glucoside) and 94.7 mg of phenols was effective in reducing the postprandial insulin response, potentially affecting the postprandial inflammatory response as suggested by the levels of hs-CRP and IL-6 [261]. In obese individuals with insulin resistance, the beneficial effects of a strawberry-based drink at 40 g at breakfast improved insulin sensitivity [260]. Concordant results were also observed in another study, where authors demonstrated that consuming strawberries (12 g) before 2 h of one meal reduced glucose concentrations and attenuated IL-6 responses [261]. The critical point approached by the papers published in the last decade dealing with dose selection is demonstrated by several papers [262,263,264]. In particular, the most recent paper is a multicenter, randomized, double-blind controlled, crossover trial on 33 obese adults (median age 53 ± 13 y; BMI: 33 ± 3.0 kg/m^2^) examining the effect of two achievable dietary doses of strawberries (13 g (one serving a day) or 32 g/day (two-and-a-half servings a day) of freeze-dried strawberry powder) on glycemic control and lipid profiles in obese adults with elevated serum LDL cholesterol. This study demonstrated that the strawberry-based beverage has a dose-dependent effect on cardiometabolic risk. The highest dose reduces both circulating insulin and insulin sensitivity, as shown by the decrease in HOMA-IR. Among adipokines, the treatment at 32 g/day significantly decreased serum plasminogen activator inhibitor-1 (PAI-1) and increased serum glucagon. However, only a borderline significant effect on serum LDL cholesterol was registered and no effects were reported for serum glucose, HbAc1 (5.5% for all treatment groups), total and HDL cholesterol, triglycerides, body weight, BMI and waist circumference [263]. 

### 5.3. Cornelian Cherry

Contrary to wild strawberries, for which in the last decade clinical trials have been more abundant than experimental investigations, the clinical evidence on cornelian cherry for glycemic control is lacking. However, the effect of the supplementation with fruit extract capsules (150mg of total anthocyanin for each tablet, four capsules/day for 6 weeks) was evaluated in a randomized, double-blind, placebo-controlled clinical trial involving 60 patients (median age 49 years) with T2DM (HbA1c between 7% and10% and duration of diabetes from at least 2 years). Compared to placebo, the extract significantly decreased HbAc1 and increased insulin levels; in parallel, a decrease in triglycerides was observed, while only a trend toward a reduction of BMI, fasting plasma glucose and 2-h postprandial glucose was registered [265]. Although these findings favor the potential use of cornelian cherry as a nutritional supplement for adult patients with T2DM, the data cannot be conclusive due to the paucity of evidence. A recruiting study aimed to determine the efficacy of 3 months of supplementation with lyophilized dried cornelian cherry on women with insulin resistance (ClinicalTrials.gov Identifier: NCT05292300) could significantly contribute to this issue.

### 5.4. Raspberry

Coming back to Rosaceae and focusing on the *Rubus* genus, red raspberry is extensively studied in the clinics. A search on the ClinicalTrials.gov database retrieved 65 results, of which 8 were related to pre-diabetes (NCT03049631 [266]), T2D (NCT03403582), blood sugar response (NCT02763020), insulin resistance (NCT02479035, NCT02479035 [267], NCT04306406), or metabolic syndrome (NCT03620617 [268], NCT01414647), aimed to test the effect of a diet rich in berries, including strawberry, raspberry and bilberry, on glucose and lipid metabolism and inflammatory marker [269,270,271].

In particular, to evaluate the effect of red raspberries consumption at breakfast in pre-diabetic women with insulin resistance (fasting glucose between 5.5 mmol/L and 7.0 mmol/L and insulin resistance value ≥ 2.5) it was performed a randomized, single-blind, three-arm, 24-h clinical [268]. The study demonstrated a reduction of the peak of both glucose and insulin and the 2-h glucose area under the curve (AUC) following 250 mg of red raspberry consumption. Similar trends of reduced insulin concentrations in response to raspberry were also observed in metabolically healthy subjects displaying obesity [267]. Similar results were obtained in a study including 25 adults with T2DM for at least 5 years, on oral hypoglycemic agents and elevated waist circumference (>89 cm for women and >102 cm for men), that demonstrated that postprandial blood glucose and the mean AUC of serum glucose significantly decreased in patients with postprandial raspberries supplementation versus control conditions, following a high-fat breakfast [272]. However, these effects were observed only in the acute phase of the study and were not confirmed after a 4-week supplementation. The trial was structured into two phases: the first postprandial phase with acute raspberry supplementation, followed by a 1-week washout and the second phase of a 10-week diet supplement with and without raspberry supplementation periods of 4 weeks each, separated by a 2-week washout phase. Despite the similarities in the acute effects of red raspberry on glycaemia and oxidized LDL, other studies report conflicting results on inflammation and oxidative stress markers [268,272]. Raspberry intake did not significantly modify postprandial inflammation and oxidative stress markers, IL-6 and IL-10. On the contrary, red raspberry reduced serum IL-6 and TNF-α in acute and chronic supplementation [273]. 

Interestingly, the titration of the frozen red raspberries revealed that among the active ingredients, 59.0% were anthocyanins (cyanidin sophoroside and cyanidin-3-glucoside in particular), followed by ellagic acid and ellagitannins (28.5%, with sanguiin H-6 the major ellagitannin) [272]. Insulin concentration was positively correlated with methylcyanidin sophoroside and negatively correlated with 4′-hydroxycinnamic acid and 4′-hydroxy-3′-methoxycinnamic acid and its isomer; no correlation was observed between circulating polyphenolic metabolites and glucose concentrations. Red raspberry at 250 mg increased the AUC_0–24h_ for methyl-cyanidin 3-O-glucoside in the pre-diabetic group [272]. Notably, it was demonstrated that ellagitannins bioavailability is dependent on the composition of gut microbiota, which could affect the clinical benefits of a supplementation base on ellagitannin-containing berries [269]. 

### 5.5. Blackberry

In 2018 Solverson et al. published the results obtained from a clinical study in which the effects of blackberry Lochness variety intake on energy substrate uses and glucoregulation in 27 volunteers of overweight or obese men (BMI > 25 kg/m^2^) consuming an HFD [274]. Subjects were fed with a controlled HFD which contained either 600 g/day blackberries (1500 mg/day flavonoids, 360 mg/day anthocyanins) or a calorie and carbohydrate matched amount of gelatin (flavonoid-free control) for seven days before a meal-based glucose tolerance test in combination with a 24 h stay in a room-size indirect calorimeter. Blackberry supplementation significantly reduced in average 24 h respiratory quotient, indicating increased fat oxidation. The respiratory quotient during the glucose tolerance test was significantly lower in patients supplemented with blackberry and a similar result was obtained in a 4 h isolation during dinner. Blackberry supplementation did not modify the glucose AUC, but the insulin AUC was significantly lower and HOMA-IR improved, with more prominent results in lower age subjects [274]. This study was conducted on a healthy subject, trials registered on ClinicalTrial.gov investigate the effects of blackberry on metabolic syndrome (NCT01944579) or T2D and gestational diabetes (NCT01474525), but their results are not yet available. 

### 5.6. Bilberry

As briefly described above, the study NCT01414647 [269,270,271] compared, in subjects with metabolic syndrome (plasma glucose 5.6–6.9 mmol/L, BMI 26–39 kg/m^2^), the effects of an 8 week of dietary supplement of 300 g of strawberry, raspberry and cloudberry against a dietary supplement of 400 g of bilberry and a standard diet (daily consumption of berries restricted to maximum 80 g). The bilberry-rich diet reduced the low-grade inflammation associated with metabolic syndrome reducing serum hs-CRP, IL-6, IL-12 and LPS and possibly triggering an immunomodulatory response as demonstrated by the regulation of the genes related to Toll-like receptors (TLR) and B-cell receptor signaling together with the expression of genes associated to monocyte and macrophage function [270]. Consistently, the blackberry diet reduced vascular cell adhesion molecule (VCAM), an immunoglobulin involved in leukocyte trafficking [275], TNF-α and adiponectin levels [276], as also confirmed by the more recent NCT02689765 study [277]. However, the same concordance was not registered in glucose metabolism: not affected in the study by de Mello et al. [271] or in the study by Chan et al. [278], in which just a slight but not significant reduction in HbA1c was observed, but in the NCT03185676 trial bilberry supplements increased both HbA1c and fasting plasma insulin [276]. However, in this latter study, the berry diet was equivalent to 100g fresh berries [276] instead of 400 g [270], a dose that, when administered for a more extended period (1-year supplementation vs. 8 weeks), improved the glucose and insulin metabolism possibly by increasing the fasting serum hippuric acid levels [271]. An apparent effect on fasting glucose was observed already after 12 weeks of 320 mg anthocyanins extracted from natural bilberry and blackcurrant administrated to subjects with newly diagnosed diabetes in the NCT02689765 study [277,279,280,281], in which a parallel increase in serum insulin-like growth factor binding protein-4 (IGFBP-4) fragment was reported. The same treatment moderately reduced HbA1c [279] and had beneficial effects on the adipocyte dysfunction associated with T2DM increasing serum adipsin and reducing visfatin [281], an adipokine whose deficiency has been associated with β-cell failure [282] firstly, and a pleiotropic protein, also known as pre-B cell colony-enhancing factor (PBEF), whose circulating levels are increased in T2DM patients [283] secondly. Similarly, the anthocyanin extract supplement at 320 mg for 24 weeks prevented insulin resistance and reduced low-grade inflammation in T2DM patients (NCT02317211 [284]). 

Collectively, these results should be interpreted in terms of both supplement dosage and duration, as underlined by Chan et al. [278], in which 1.4 g extract (containing ≥ 25% anthocyanidins)/day were administered but for just 4 weeks. The recruiting study NCT04637945 was designed to evaluate the effect of 8 weeks of supplementation with a mixture of anthocyanin from bilberry, black currant extract and black rice on glucose homeostasis and inflammation should contribute to the debate. However, the study by Hoggard et al. [285], registered as NCT01180712 trial, demonstrated in subjects with T2DM controlled by diet and lifestyle alone or with impaired glucose tolerance that the supplementation with a standardized bilberry extract (Mirtoselect^®^, 36% anthocyanins and equivalent to 50 g of fresh bilberries) at 0.47 g acutely lower the incremental plasma glucose AUC by 18% compared with the placebo. The extract did not affect plasma glucose-dependent insulinotropic polypeptide (gastric inhibitory polypeptide, GIP), glucagon-like peptide 1 (GLP-1), glucagon, or amylin, as well as the inflammatory adipokine monocyte chemoattractant protein 1 (MCP-1). These results led the authors to hypothesize that these acute effects could deal with the polyphenols content other than anthocyanins (about 18%) which could reduce carbohydrate digestion and absorption (NCT01245270 [285]). The recruiting studies NCT04004182 could probably contribute to defining bilberry’s contribution to inhibiting intestinal glucose transporters. Nevertheless, the high phenolic content of not only bilberry but also rosehip was associated with the reduction in the very early phase (0–30 min) of postprandial insulin response (NCT03159065 [286]). These promising results should be extended in the long-term study NCT03185676, completed in 2017, but whose results have not yet been widespread. Finally, the beneficial effect of bilberry on diabetic retinopathy is also under investigation. Apart from the NCT02984813 study, oral supplementation with Mirtoselect^®^ was tested in T2DM patients with severe pre-proliferative retinopathy with a preventive effect on the worsening of retinopathy symptoms [287], confirming the potential of this supplement in the retinopathies [288], diabetic included.

### 5.7. Rosehip

Apart from the NCT03159065 trial, investigating the comparative effect of different supplements, rosehip included, no other trials dialing with anti-diabetic properties of this plant are reported on the ClinicalTrial.gov database. However, the combinatory effect of a standardized extract from seven plants, *Rosa canina* L. (225mg), *Capparis spinosa* L. (170 mg), *Securidaca securigera* L. (170 mg), *Silybum marianum* L. (65 mg), *Urtica dioica* L. (170 mg), *Trigonella foenum-graecum* L. (115 mg) and *Vaccinium arctostaphylos* L. (85 mg), was recently investigated in T2DM patients (men and woman 40 to 60 years old with fasting plasma glucose level from 130 to 160 mg/dL and HbA1c 7.5% to 8.5% and under the therapy of oral anti-hyperglycemic drugs maximum 10 mg glyburide and 1000 mg metformin daily) [289]. The study was designed as a three-arm (herbal composition, metformin and placebo), double-blind, randomized placebo-controlled clinical trial. The interventions herbal combination (1000 mg), metformin (250 mg), or placebo were administered once a day before the meal. The authors showed a significant decrease in fasting plasma glucose and HbAc1 compared to the placebo, but no differences were observed in lipid outcomes, renal, hepatic and hematological serum parameters. These results are only partially in accord with the data from previous studies [230]. A randomized, double-blind placebo-controlled trial involving 60 patients with T2DM (aged 35–60 years with fasting blood glucose levels between 130 to 200 mg/dL and HbA1c between 7–9% despite using conventional oral hypoglycemic drugs) compared the effect of 750 mg rosehip fruit extract to placebo in a 3-month treatment [230]. The long-term treatment was able to significantly reduce both fasting blood glucose and cholesterol/HDL-C ratio. However, no significant differences were measured for postprandial blood glucose, HbA1c, total cholesterol, triglyceride, LDL-C/HDL-C ratio, blood urea nitrogen (BUN), creatinine, serum glutamic-oxaloacetic transaminase (SGOT) and serum glutamic pyruvic transaminase (SGPT). The effects on glucose and lipid profile observed in the present study in part may be due to phenolic compounds present in rosehip [290]. Another randomized, double-blind, cross-over study involving 30 obese subjects with normal or impaired glucose tolerance, showed that a daily intake of a rosehip powder drink over 6 weeks, compared with a control drink, did not induce significative changes in glucose tolerance, plasma levels of HDL cholesterol, triglycerides, incretins and markers of inflammation. However, the authors observed a significant systolic blood pressure and LDL/HDL ratio reduction in the treatment group. In particular, the rosehip drink assumption significantly reduced the Reynolds risk assessment score for cardiovascular disease, thus suggesting that the rosehip extract could reduce the risk markers of T2DM and cardiovascular disease [291].

## 6. Discussion

Although the use of herbal remedies in developed countries is growing [292], many challenges must still be faced before validating them as both functional food and eventually effective drugs. First, the traditional knowledge of local plants should be reinterpreted based on modern science [293]. This more and more general approach can also be applied to the linguistic islet minority, where traditions are usually well settled but linked to oral transmission and customary use with the evident risk of getting lost. The process of traditional knowledge reinterpretation according to modern science could have evident positive effects, which include the economic development of the regions to which the specific herbal tradition belongs, the preservation of the cultural identity of linguistic islets minority and, more importantly, the promotion of a culture of traditional herbal remedies based on evidence. This latter aspect is in line with the World Health Organization’s (WHO) strategy 2014–2023, which aimed to strengthen the role of traditional medicine and promote the utilization of medicinal plants in the different health systems of its member countries [278].

In our analysis, we evaluated the last 10 years of evidence-based data on the potential anti-diabetic properties of nine plants used in the Occitan valley of the Piedmont Alps. Notably, the local uses of the plants described are not related to diabetes and only a few of them have been traditionally used with this indication in other countries, such as yarrow in Peru [66], rosehip in Turkey [227], or bilberry in Russia [294]. The literature points out experimental and clinical evidence suggesting their anti-diabetic properties. However, before a consensus could be reached, a rigorous and quantitative analysis of their consumption should be performed. This data should be cross-linked with the prevalence and diffusion of T2DM in the selected geographical area to which the traditional heritage belongs. Unfortunately, this data is unavailable, thus affecting any conclusion on the preventive role of the consumption of the plants herein reported.

### 6.1. Herbal Remedies for Diabetes: The Bench-to-Bedside Challenge

Herbal remedies could be focused on different therapeutic anti-diabetic approaches: (1) the prevention of T2DM by consuming plants, for which the traditional uses are primarily as food; (2) supplementation strategy as an add-on treatment to potentiate the effect of the current anti-diabetics; (3) use the plant extracts as anti-diabetics. 

The first two strategies converge on the concept of functional foods. They are the most reliable, at least nowadays, as demonstrated by the current clinical studies in this review evaluating the use of herbal remedies as add-on therapy for T2DM or metabolic syndrome. Notably, the mechanism(s) of glycemic control shown by the plants considered in our study converges on the ability to reduce intestinal glucose absorption (Table 2 and Figure 1). Therefore, they could be an exciting option in pre-diabetic patients’ borderline for T2DM development, for which no specific drugs are currently approved. Under this task, evaluating the effect of herbal remedies on risk factors for T2DM development, inflammation, obesity and lipid profile, including [295], is desirable. 

On the contrary, the use of the plant extracts as anti-diabetics is still far from being proven, even if some experimental studies herein reported demonstrated that several extracts had a similar effect to acarbose [110,210], glibenclamide [103,105,108,128,232,251], or even metformin [192,251,289]. However, appropriate non-inferior or, even better, superiority studies and comparative clinical trials are mandatory to assess the anti-diabetic effect and the therapeutic positioning. 

The reported data indicates that both preclinical and clinical studies are ongoing for almost all the selected plants. However, the majority are preclinical investigations. In vivo studies rely primarily on an experimental design that mimics T1DM, using the STZ-induced diabetes model. This is the most reproducible and consistent model of diabetes. However, it mimics T1DM, as STZ destroys the β cells leading to a dramatic deprivation of insulin [296,297]. Some other authors used alloxan-induced diabetes, again a model of insulin-dependent diabetes [298]. Due to these models based on pancreatic damage could help evaluate the protective effect of the natural extract on β cell integrity, as reported for yarrow [77,103] or red raspberry [110], but do not produce results completely transferable on T2DM. 

T2DM accounts for 90% of diabetes cases and has a multifactorial etiology, where lifestyle and diet have a pivotal role. T2DM models could validate specific herbal remedies as a preventive or add-on therapy. Therefore, implementing studies using HDF or STZ+nicotinamide models, both reproducing T2DM [299,300], should be encouraged. Moreover, a stricter indication of the supplement initiation should also be included in all the experimental studies, thus clarifying the prophylactic or therapeutic design and improving the transfer from the bench to the bedside of the results obtained.

Overall, we must recognize that the studies published between 2011–2021 present several limitations that necessary affect our review. Basically, their quality is sub-optimal, for instance, a proper qualitative and quantitative characterization of the extract use is often missing, as well as the part of the plant utilized sometimes not reported. Moreover, the experimental reports mostly rely on endpoints that do not allow efficacy extrapolation and many studies only replicate and confirm previous data, eventually pointing out plants of different variety, genotype, storage, processing, climate, soil and harvest time or remedy preparations (i.e., aqueous or ethanol extract), but again the papers usually do not specify the cultivar differences and are not comparative. 

Looking at the clinical studies, evaluating any efficacy is still far from conclusive. The number of studies is limited and the quality is affected by the number of individuals included and the absence of a stratification considering the co-variables that could occur (such as sex, age, duration of diabetes, anti-diabetic and non-anti-diabetic drugs, BMI at baseline, co-morbidities).

### 6.2. Herbal Remedies for Diabetes: The Standardization of Titration Challenge

Apart from the experimental design, the challenge of understanding the actual contribution to diabetes management of the plants in this review (but for all plants in general) deals with many issues, starting from the several different preparations that could be obtained and including extraction, fractionation, purification, concentration, fermentation and distillation [99]. All of these could have different active ingredient titrations that make difficult any attempt at standardization. This could be affected by the production process reported for raspberry. Their anthocyanins content could decrease by 42% in frozen berries compared to their raw counterparts [230]. Differences in the raw material account for the extract titration variability. This point is even more critical when considering the differences between cultivars’ conditions. Variety, genotype, storage, processing, climate, soil and harvest time affect the phenolic composition of plants [82]. A comparison of nutritional and nutraceutical properties in wild strawberry fruits cultivated in Italy evidenced that in the same region, at the same altitude and under the same agricultural practices, the genotype exerts a significant effect on the synthesis of polyphenols: higher in *Sara* and *Alpine* than in *Regina delle Valli* and *Valitutto* varieties [301]. Thus, underling how the specific variety could affect the results in active ingredients should be declared in the experimental design to allow comparison and obtain conclusive data. Interestingly, higher amounts of sugar and organic acid were found in wild strawberries grown at the highest altitude despite the altitude did not affect the secondary metabolites and radical scavenging activity [302]. According to the organ or the plant development stage, the sugar composition of some polysaccharides or the proportion of substituents linked to sugar could be affected. These differences could significantly affect the active ingredients’ biological activity and the whole plant. It has been reported that the yield of oligosaccharides differs based on the plant’s growth level, which affects the anti-coagulant activity [174]. Moreover, looking at oligosaccharides, their standardization is challenged by isolating individual oligosaccharides with characterized structures [303]. The isolation and chemical characterization of unique active ingredients are one of the most dramatic issues. Indeed, the literature generically reports phenols, polyphenols, anthocyanins and tannins without grouping them correctly. Proper identification and quantification of bioactive constituents should be necessary to predict the biological properties of extracts of plants [29]. 

Even when extracts are enriched in one of the active ingredients, such as anthocyanins, it is unlikely this should be the only active ingredient present. The role of the single active ingredient is based on the relative amount compared to the others present. Therefore, it is possible to argue that anti-diabetic effects are the results of the synergism of all the components of the extract. We identified as major actors in the described anti-diabetic effects, several substances summarized in polyphenols and oligosaccharides. However, also vitamins, carbohydrates, minerals and dietary fibers are present and their contribution to the whole extract effect should not be ruled out. For instance, dietary fibers, affecting the digestive processes could indirectly reduce the glycemic index [304]. The complex mechanism includes the selective promotion of gut bacteria [305]. These issues inevitably bring out the bias generated using different extracts without rigorous standardization, which makes the comparison between studies just elusive and not conclusive. Nevertheless, other studies not included in this review used more than one plant as an active treatment, thus making it even more challenging to dissect each plant’s relative contribution and the functional role of each active ingredient. Even if titration standardization is solved, bioavailability should still be considered.

### 6.3. Herbal Remedies for Diabetes: The Bioavailability Challenge

The chemical structure of polyphenols can influence their bioavailability, as their binding to sugars (as glycoside) can increase solubility. Additionally, polyphenols must be released from the food matrix to be absorbable after oral administration. Intestinal absorption can be influenced by intestinal enzymes and colonic microflora [262], which hydrolyze polyphenols that the native form cannot absorb (polymers, esters, glycosides). Due to the low permeation through the intestinal barrier, the amount of bioactive compound in the bloodstream is often very low. They also undergo complex metabolism in the gut and liver [306]. In this regard, the effect of gut microbiota is not negligible. For instance, it was demonstrated that ellagitannin bioavailability is dependent on the composition of gut microbiota, which could affect the clinical benefits of a supplementation base on ellagitannin-containing berries [270]. Indeed, ellagitannins are not absorbed in the small intestine but in the colon, where microbiota converts them to urolithins [307].

Notably, a bidirectional interaction exists: the ellagitannin-rich berry consumption stimulated a predominance of Ruminococcaceae and Lachnospiraceae [269]. Consistently, gallic acid had been reported to enhance the number of *Atopobium* spp. Bacteria and reduced that of Clostridium histolyticum and anthocyanins increase *Bifidobacterium* spp. and *Lactobacillus-Enterococcus* spp. [308,309]. Similarly, cornelian cherry promotes the development of prebiotic microorganisms with a similar detrimental effect on the growth of potentially pathogenic microorganisms [310,311]. This reciprocal influence further supports the hypothesis of a prominent gastrointestinal activity of herbal plants that could inhibit the α-glucosidase enzyme before systemic absorption and, in parallel, improve the microbiota system contributing to a “healthy” gut microbiota [312]. On the other side, this data points out the possible inter-individual variability in bioavailability and consequently in response to dietary interventions [313].

The problem of sub-optimal bioavailability deals with the high dosages requested to obtain the desired effects. It is not unusual that the most active polyphenol is not the most bioavailable [314]. For instance, the number of bilberries eaten for a clinical effect corresponded to 400 g of fresh berries daily [271]. As reported by the authors, the average intake of berries in the Finnish population on which the study was conducted is 50–70 g/day. Increasing dosages could lead to the onset of toxicity. Overconsumption of polyphenols could have hypoglycemic effects [5]. Therefore, their overconsumption could dramatically cause a pharmacodynamic interaction with hypoglycemic agents. Pharmacodynamic interaction can also occur beyond the hypoglycemic effect: for instance, *Vaccinium Mirtillus* L. could increase bleeding in patients with anti-coagulants treatment [315]. In parallel, pharmacokinetics interaction could be overcome. Therefore, monitoring the acute and long-term side effects of natural compounds and their interaction with food and drugs is strictly needed. The 400 g/day of bilberries could be realistically obtained only using freeze/dried berries [232], which could be lower concentrated in active ingredients. 

To increase polyphenol bioavailability and protect them from metabolism, several strategies could be considered, including condensed forms. The new frontiers focus on natural products encapsulation in nanocarriers, which also allow to control and target their release [316]. An example is the use of gold nanoparticles functionalized with elderberry extract tested in a model of STZ-induced diabetes [317]. This formulation was more active than elderberry extract in reducing the HbA1c. However, new formulations based on nanoparticles deserve deep and long-term studies to evaluate the safety profile.

## 7. Conclusions

This review reveals scantly sparse literature, with plants growing in different areas and under other conditions, without rigorous standardization. Nevertheless, specific variety from the linguistic ‘isles’ of Piedmont has never been tested for their anti-diabetic properties. However, the lack of homogeneity in the provenience of the plants and the consistency among the results obtained favor a generalization of the evidence collected. Therefore, we can conclude that using the selected Alpine plants belonging to the “linguistic isles” of Piedmont as a functional food for T2DM is reasonable. The most vital data are experimental, but the clinical assessment is ongoing. However, studies on single varieties grown in the geographical area, with strict standardization and titration of all the active ingredients, are warranted before applying the WHO strategy 2014–2023.

## Figures and Tables

**Table 1:** Plants used in the Occitan valleys of the Piedmont Alps for which anti-diabetic properties have been suggested by the scientific literature published between 2011–2021.

Family	Botanical Name	Plant	Altitude (msl)
Adoxaceae	*Sambucus nigra* L.	Black elderberry	0–1400
Asteraceae	*Achillea millefolium* L.	Yarrow	0–2500
Cornaceae	*Cornus mas* L.	Cornelian cherry or dogwoods	0–1500
Ericaceae	*Vaccinium myrtillus* L.	Bilberry	1200–2000 (rarely 300–2800)
Rosaceae	*Fragaria vesca* L.	Wild strawberry	200–1900 (rarely up to 2400)
Rosaceae	*Rosa canina* L.	Rosehip or dog rose	0–1900
Rosaceae	*Rubus fruticosus* L., *Rubus ulmifolius* L.	Blackberry	0–1400
Rosaceae	*Rubus idaeus* L.	Raspberry	200–2000
Urticaceae	*Urtica dioica* L.	Stinging nettle	0–1800

msl = meters above sea level.

**Table 2:** Plants used in the Occitan valleys of the Piedmont Alps: potential direct anti-diabetic mechanisms (experimental evidence between 2011 and 2021).

Plant	Part of the Plant Tested		Mechanisms (Experimental Evidence)	References
	Formulation	Identified Active Ingredients		
*Urtica dioica* L.	Leaves		insulin secretion (++) insulin sensitivity (++) α-glucosidase/α-amylase inhibition (+)	Ahangarpour et al., 2012 [91] Dar et al., 2013 [92] Kadan et al., 2013 [93] Qujeq et al., 2013 [94] Rahimzadeh et al., 2014 [95] Ranjbari et al., 2016 [96] Obanda et al., 2016 [97] Obanda et al., 2016 [98] Gohari et al., 2018 [99] Abedinzade et al., 2019 [100] Fan et al., 2020 [101] Salim et al., 2020 [102]
	H_2_O extract [92,94,95,96]	polyphenols (phenolic acids, flavonoids and anthocyanins)		
	Hexane extract [92]	terpenes, fatty acids [92]		
	Chloroform extract [92]			
	Ethyl-acetate [92]			
	MeOH [92]			
	EtOH extracts [94]			
	Hydroalcoholic extract [91]			
	Leaves and stem			
	Hydroalcoholic extract [93]			
	Aerial parts			
	Hydroalcoholic extract [100]			
	powder [101]			
	distillate [99]			
	H_2_O extract [97,98]			
*Achillea millefolium* L.	Aerial parts		insulin secretion (++) insulin sensitivity (++) α-glucosidase inhibition (+)	Mustafa et al., 2012 [103] Ramirez et al., 2012 [104] Zolghadri et al., 2014 [77] Chávez-Silva et al., 2018 [105]
	H_2_O extract [103]	Tannins, glycosides, terpenoids, flavonoids and phenolics [103]		
	MeOH extract [103]			
	Hydroalcoholic extract [77,104,105]			
*Vaccinium myrtillus* L.	Leaves with stems		insulin secretion (+/-) α-glucosidase inhibition (+) α-amylase inhibition (+/-) reduction of glucose absorption (+)	Brader et al., 2013 [106] Kim et al., 2015 [107] Asgary et al., 2016 [108] Buchholz & Melzig, 2016 [109] Bljajić et al., 2017 [110] Karcheva-Bahchevanska et al., 2017 [111] Xiao et al., 2017 [112] Schreck & Melzig, 2021 [113]
	H_2_O extract [110]	Polyphenols: phenolic acids > flavonoids [110]		
	Hydroalcoholic extract [110]	Polyphenols: flavonoids > phenolic acids [110]		
	Fruits			
	MeOH:H_2_O:trifluoroacetic acid extract [106]	Polyphenols (anthocyanins, phenolic acids, flavonols) [106]		
	Hydroalcoholic extract [107,112]	Polyphenols (anthocyanins, flavonoids and phenolic acids) [112] Anthocyanins [107]		
	MeOH:H_2_O: HCl [111]	Polyphenols [111]		
	Acetone: H_2_O: HCl [111]			
	H_2_O extract [109,111,113]			
	MeOH extract [109,113]			
	Powder [108]			
*Rubus idaeus* L.	Fruits		insulin sensitivity (+) α-glucosidases/α-amylase inhibition (+)	Zhu et al., 2018 [114] Xiong et al., 2018 [115] Zhao et al., 2018 [116] Xing et al., 2018 [117] Gutierrez-Albanchez et al., 2019 [118]
	Powder [114,116,117]	Polyphenols [114,116]		
	MeOH extract [118]	Polyphenols (anthocyanins) [118]		
*Rubus fruticosus* L., *Rubus ulmifolius* L.	Fruits		insulin sensitivity (+/-)	Bispo et al., 2015 [119] Gowd et al., 2018 [46]
	MeOH extract [119]			
	EtOH extract [46]			
*Fragaria vesca* L.	Leaves		α-glucosidase/α-amylase inhibition (+)	Takács et al., 2020 [120]
	H_2_O extract [120]	Flavonoids [120]		
*Sambucus nigra* L.	Flowers		insulin sensitivity (+/-) α-glucosidase/α-amylase inhibition (+)	Schrader et al., 2012 [121] Bhattacharya et al., 2013 [122] Farrell et al., 2015 [123] Salvador et al., 2016 [124] Ho et al., 2017 [125] Ho et al., 2017 [126] Zielinska-Wasielica et al., 2019 [127]
	MeOH extract [121,122]	Polyphenols (phenolic acids, flavonoids) [122] Polyphenols [126]		
	DCM extract [122,126]			
	EtOH extract [126]			
	Hydroalcoholic extract [126]			
	H_2_O extract [126]			
	Fruits			
	Anthocyanin-rich extract [123]	Anthocyanins [123]		
	DCM extract [125]	Polyphenols (anthocyanins and procyanidins) [125]		
	EtOH extract [125]			
	Hydroalcoholic extract [125]			
	H_2_O extract [125]			
	pressed juice [125]			
	H_2_O extract [127]	Polyphenols (anthocyanins, flavonoids, phenolic acids) [127]		
*Rose canina* L.	Pseudo fruits and flowers		insulin secretion (++) insulin sensitivity (+/-) α-amylase inhibition (+)	Taghizadeh et al., 2016 [128] Fattahi et al., 2017 [129] Jemaa et al., 2017 [130] Chen et al., 2017 [131] Bahrami et al., 2020 [132] Rahimi et al., 2020 [133]
	MeOH extracts [130]	Flavonoids [130]		
	Pseudo fruits			
	Oligosaccharide fraction of extract [132,133]	Oligosaccharides [132,133]		
	H_2_O extract [129,131]			
	Hydroethanolic extract [128]			
*Cornus mas* L.	Fruits		insulin sensitivity (+/-) α-glucosidase/α-amylase inhibition (+)	Capcarova et al., 2019 [134] Dzydzan et al., 2019 [135] Dzydzan et al., 2020 [136] Blagojevic et al., 2021 [137]
	Pressed juice [25,136]	Polyphenols (anthocyanins, phenolic acids, flavonols) and iridoids (loganic acid) [135] Iridoids [136]		
	Hydroalcoholic extract [137]	Iridoids and anthocyanins [137]		
	Homogenized [134]			

DCM: dichloromethane; H_2_O: water; MeOH: methanol; EtOH: ethanol; ++ = in vitro and in vivo consistent results; + = in vivo or in vitro data; +/- = studies with contrasting results.
